# Designing exotic many-body states of atomic spin and motion in photonic crystals

**DOI:** 10.1038/ncomms14696

**Published:** 2017-03-08

**Authors:** Marco T. Manzoni, Ludwig Mathey, Darrick E. Chang

**Affiliations:** 1 ICFO-Institut de Ciencies Fotoniques, The Barcelona Institute of Science and Technology, 08860 Castelldefels (Barcelona), Spain; 2 Zentrum für Optische Quantentechnologien and Institut für Laserphysik, Universität Hamburg, 22761 Hamburg, Germany; 3 The Hamburg Centre for Ultrafast Imaging, Luruper Chaussee 149, Hamburg 22761, Germany

## Abstract

Cold atoms coupled to photonic crystals constitute an exciting platform for exploring quantum many-body physics. For example, such systems offer the potential to realize strong photon-mediated forces between atoms, which depend on the atomic internal (spin) states, and where both the motional and spin degrees of freedom can exhibit long coherence times. An intriguing question then is whether exotic phases could arise, wherein crystalline or other spatial patterns and spin correlations are fundamentally tied together, an effect that is atypical in condensed matter systems. Here, we analyse one realistic model Hamiltonian in detail. We show that this previously unexplored system exhibits a rich phase diagram of emergent orders, including spatially dimerized spin-entangled pairs, a fluid of composite particles comprised of joint spin-phonon excitations, phonon-induced Néel ordering, and a fractional magnetization plateau associated with trimer formation.

## Abstract (web summary)

Cold atoms coupled to photonic crystals constitute a platform for exploring many-body physics. Here the authors study the effect of coupling between the atomic internal degrees of freedom and motion, showing that such systems can realize extreme spin-orbital coupling and uncover a rich phase diagram.

Rich phenomena in condensed matter arise when quantum spin systems couple to phonons or orbital degrees of freedom of the underlying crystal lattice. Perhaps the most famous example is the spin-Peierls model^1^,^2^,^3^,^4^, wherein the spin interaction leads to a lattice instability resulting in a ground state of singlet pairs and a bond-ordered density wave. Motivated by this emergence of new physics, it is tempting then to consider the most extreme limit of coupling between spin and motion—where the spin-carrying particles are completely free to move, and the spin-dependent forces thus dictate the properties of the emergent spatial order. Such a material would constitute a novel ‘quantum crystal' that has not existed before, in which the emergent spatial patterns and spin properties are intricately locked together, and where driving one would automatically affect the properties of the other.

One possible route toward realizing such a material involves the new experimental platform consisting of cold atoms coupled to photonic crystal structures. Photonic crystals^5^ are periodic dielectric structures in which the propagation of light can differ significantly from uniform media. An important feature of photonic crystals is the appearance of photonic band gaps, where strong interference in scattering from the periodic dielectric yields a complete absence of propagating modes within some bandwidth. Nominally, an excited atom whose transition frequency resides in the gap would not be able to spontaneously emit; instead, it has been shown that an atom-photon bound state can form, in which the atom becomes dressed by a localized photonic cloud^6^,^7^,^8^,^9^,^10^. The tight spatial confinement associated with this photon yields large dispersive forces on proximal atoms that depend on the atomic internal ‘spin' states, which thus realizes the required spin-dependent forces to possibly observe the phenomena described above.

In this paper, we first describe the key features of atoms coupled to photonic crystals. While this interface in principle enables the realization of many different Hamiltonians^9^,^10^,^11^, we focus on one model where atoms are trapped in a weak one-dimensional external potential, and where a short-range spin-dependent force can be made sufficiently strong to exceed the external potential. To understand the emergent orders of this system, we begin by treating the motion of the atoms classically and their spins quantum mechanically. We find an effect reminiscent of the spin-Peierls transition, in which the atoms spatially dimerize and realize a high degree of entanglement within each dimer. We then proceed to a fully quantum model. Using density matrix renormalization group (DMRG), we find a rich variety of quantum phases beyond the spin-Peierls state, such as a state where spin and phonon excitations form composite particles, phonon-induced Néel ordering, and spatial trimers associated with magnetization plateaus. While here we study a specific model to create correlated spin-orbital quantum matter, more generally this work suggests that spin-orbital coupling can be a dominant phenomenon in all hybrid systems of atoms and photonic crystals. Similar considerations could also apply to a number of other atomic systems where spatially-dependent spin interactions can be realized, including polar molecules^12^,^13^,^14^, Rydberg atoms^15^, ion chains^16^,^17^,^18^, and atoms in high-finesse cavities^19^.

## Results

### Atoms coupled to photonic crystal waveguides

**Figure 1:**
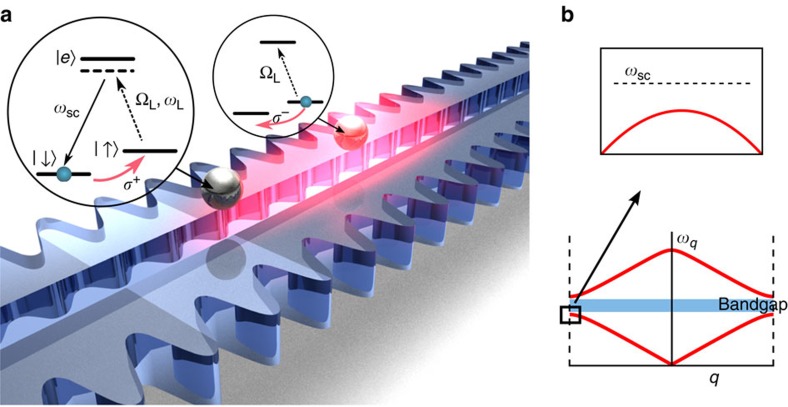
Atoms interacting with a photonic crystal waveguide. (**a**) Schematic rendering of the ‘alligator' photonic crystal waveguide^20^ with two atoms trapped. The atomic transition 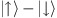 is globally driven by an external laser with Rabi frequency Ω_L_. In principle, an atom originally in 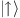 can Raman scatter a laser photon and flip to state 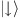. However, when the frequency of the scattered photon *ω*_sc_ lies within a bandgap (see **b**), this photon becomes bound around the atom (illustrated by the pink cloud). It can be subsequently absorbed by another atom initially in state 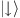, resulting in a flip to state 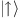. (**b**) Illustration of the dispersion relation (frequency *ω*_*q*_ versus Bloch wavevector *q*) of the guided modes. The scattered photon frequency *ω*_sc_ is aligned to a bandgap where no guided modes exist.

Photonic crystals^5^ are periodic dielectric structures in which the propagation of light can differ significantly from uniform media (Fig. 1a). The dispersion relation in such structures consists in general of different bands, between which can appear bandgaps—frequency regions in which the light cannot propagate inside the crystal (Fig. 1b). Particularly rich phenomena are predicted to arise when an atomic transition is driven by a laser at a frequency within the bandgap. A specific example is illustrated in Fig. 1a, where two identical atoms are coupled to an ‘alligator' photonic crystal waveguide (PCW), which consists of two separate waveguides whose modes hybridize with one another. Atoms have recently been coupled to such a structure in experiments described in refs 20, 21, 22, 23. Here, the atoms are assumed to have three relevant electronic levels, with two ground (or metastable) states 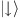, 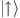 connected by a common excited state 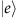. The transition between ground state 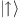 and excited state 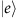 is globally driven by an external laser with frequency *ω*_L_ and Rabi frequency Ω_L_, while the transition between ground state 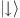 and 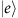 is coupled to the guided modes of the waveguide. In principle, an atom in state 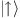 could Raman scatter a pump photon into the waveguide and flip to state 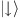. However, when the frequency *ω*_sc_=*ω*_L_+*ω*_↑_−*ω*_↓_ of that scattered photon lies within the bandgap (see inset of Fig. 1b), it is unable to propagate and instead forms a bound state of length *L* around the atomic position. A second atom nearby in state 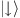 can subsequently absorb that photon, resulting in an effective spin flip interaction between the two atoms^9^. The effective spin Hamiltonian, generalized to many atoms, takes the form


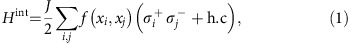


with 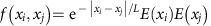. *σ*^−^=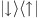 denotes the spin lowering operator from 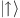 to 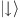, and conversely for *σ*^+^. *J* and *L* are the strength and characteristic length of the interaction respectively, which are tunable through the laser parameters *ω*_L_, Ω_L_. *E*(*x*_*i*_)=cos *kx*_*i*_, with *k*=*π*/*a* (where *a* is the lattice constant of the PCW), is the Bloch function associated with the electric field at position *x*_*i*_. A more detailed microscopic derivation of the effective Hamiltonian (1) based on ref. 9 is provided in Supplementary Note 1 (also see Supplementary Fig. 1). Here, we will assume that the atoms are tightly trapped in the transverse directions such that the position along *x* is the only dynamical variable. Absent any motional effects (i.e., if *f* is constant), equation (1) corresponds to the ‘XX' spin model in 1D^24^.

The possibility to tune *J*, *L* and even the type of spin interaction makes the atom-photonic crystal interface a promising candidate for the simulation of many-body spin models with long-range interactions, when atoms are trapped at fixed positions^9^,^10^,^11^. Beyond photonic crystals, there are also a number of other proposed approaches to realize spin models with atoms^14^,^16^,^17^,^18^,^25^,^26^,^27^. Here, our goal is to investigate phenomena that can occur when motion is included as well. In particular, if one treats the position variables *x*_*i*_ as dynamical degrees of freedom, the Hamiltonian in equation (1) should be regarded as a spin-dependent potential, wherein the forces experienced by the atoms can depend on the spin correlations. As these forces originate from the dispersive forces associated with photons confined to the nanoscale, their magnitude can be comparable to or much larger than those associated with conventional optical trapping forces^9^, implying that the physics of spin-motion coupling can become prominent in such systems. For example, using an electronic transition in a typical alkali atom, *J* can approach the GHz scale, as compared to ∼MHz scales for the excited state spontaneous emission rate and external trap frequencies (see Supplementary Note 1 for details). In emphasizing the role of spin correlations on motion, we also extend previous ideas involving self-organization of atoms in cavities or waveguides due to optical forces, where the atoms are treated essentially as classical dielectric particles with no internal degrees of freedom^28^,^29^,^30^,^31^,^32^,^33^.

### Classical motion

**Figure 2:**
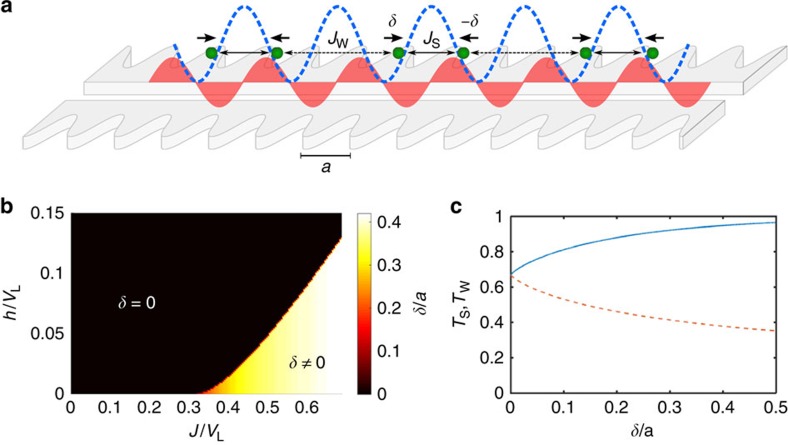
Spin-motion coupling in the limit of classical motion. (**a**) Schematic 1D representation of the model, with atoms (green) trapped in an external potential (blue). The photonic-crystal mediated interaction is modulated by the standing wave of the Bloch modes (red), while the external potential creates trapping sites centred around the nodes. The arrows represent the displacement from the trapping sites to a dimerized configuration. (**b**) Spatial dimerization *δ* (in units of the lattice constant *a*), as a function of the interaction strength *J* and the magnetic field energy *h* (in units of the external trap depth *V*_L_). (**c**) Triplet fraction of the reduced density matrix for two atoms within a dimer (*T*_S_, blue solid curve), and consecutive atoms in different dimers (*T*_W_, red dashed), as a function of dimerization *δ*, at zero magnetic field (*h*=0).

We propose a realistic experimental setup, which highlights the interplay of spin and motion. Atoms interact via the Hamiltonian of equation (1), and are separately trapped by an external, spin-independent optical lattice 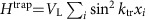 (this could originate from optical fields in another guided band far from the atomic resonance). Peculiarly, this lattice traps atoms at the nodes of the Bloch function, and thus nominally hides the atoms from the PCW interaction. Despite not being a fundamental requirement to see spin-motion coupling, we assume that the trapping wavelength is such that atoms are localized around even nodes of the Bloch wave functions, i.e., *k*_tr_=*k*/2=*π*/(2*a*), where *a* is the length of the unit cell of the PCW, as pictured in Fig. 2a. It can be readily shown that within our model, trapping atoms at every site would yield a phase transition with discontinuous change in the atomic positions.

We consider the Hamiltonian in the case of one atom per trapping site and an external magnetic field that can polarize the atoms with energy *h* along *z*:


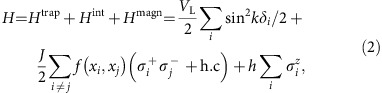


where *δ*_*i*_ denotes the displacement of atom *i* from the bottom of its external well. In the present section we treat the atomic position classically, while investigating the case of quantum motion in the next section. We assume that the coupling strength *J* is positive. For simplicity, in equation (2) we also ignore the self-interaction term (*i*=*j*), which can be compensated by an external potential.

To study the many-body ground state of Hamiltonian (2) without any assumption about the spatial configuration is very difficult. Furthermore, for 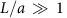 the long-range character of the interaction makes the spin model relatively difficult, even for fixed positions. As a consequence, we restrict our attention to the case *L*∼*a*, for which we can make a nearest-neighbor approximation. We can get an intuition of the possible ground state configuration of a system of many atoms by considering how just two atoms in neighboring sites interact. If the atoms remain at the bottom of their trapping wells, the function *f*(*x*_1_, *x*_2_)=0 as these positions coincide with nodes of the Bloch functions. However, the PCW interaction energy would become negative, if the two atoms were to form a triplet state, 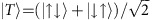 (or a singlet for *J*<0), and simultaneously displace toward each other to form a spatial dimer. Such a process would become energetically favourable overall for a certain ratio of *J*/*V*_L_. Motivated by this simple case we make an ansatz that the spatial configuration of the many-body ground state consists of dimerized pairs. In particular, we assume that *x*_*i*_=2*ia*+(−1)^*i*^*δ*, where *δ* represents the displacement from the trap center, as pictured in Fig. 2a. This is reminiscent of the lattice instability that creates entangled dimers in the spin-Peierls model^1^, but with the substantial difference that our system becomes non-interacting in the absence of dimerization (as the atoms are at the nodes). In the following, we treat *δ* as a variational parameter and proceed to solve the spin ground state exactly.

The nearest-neighbor spin Hamiltonian can be mapped to a chain of spinless fermions through standard Jordan-Wigner transformation^34^, with the presence/absence of a fermion on a site corresponding to spin up/down, respectively. Because of the staggered spatial configuration, it is natural to define a unit cell *j* consisting of a pair of dimerized atoms (labelled L,R). Two different spin couplings 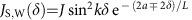 then characterize the interaction between atoms within the same dimer, and between consecutive atoms *R*,*L* in neighboring dimers, respectively (see Fig. 2a). The Hamiltonian then reads


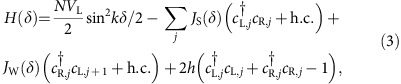


where *c*_(L,R),*j*_ are fermion annihilation operators for site *j*. Just as in the standard Jordan-Wigner transformation, this two-spin per-site Hamiltonian can be exactly or numerically diagonalized by moving to Fourier space, as we describe in Supplementary Note 2.

By minimizing the ground-state energy of *H*(*δ*) with respect to *δ* we find the optimal spatial configuration (within the ansatz). In Fig. 2b we plot the resulting value of *δ* as a function of the interaction strength *J* and of the magnetic field *h* (in units of *V*_L_). In the *J*−*h* plane one can clearly distinguish a critical value of the spin interaction strength, *J*_crit_(*h*), above which a phase transition occurs from a non-interacting to a dimerized state. The increase in spin entanglement with dimerization can be quantified by taking the two-particle reduced density matrix *ρ*_2S_ of atoms within a dimer, and calculating its overlap with the triplet state, 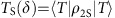. We plot *T*_S_(*δ*) in Fig. 2c for zero magnetic field. For *δ*=0 this quantity tends to the value in the conventional XX spin model, *T*_S_(0)=(1/2+1/*π*)^2^≈0.67, while for large values of *δ* and small *L* it tends to 1. Similarly, defining an analogous quantity *T*_W_(*δ*) between consecutive atoms in neighboring dimers, we find a decrease in correlation with increasing dimerization.

### Quantum motion

**Figure 3:**
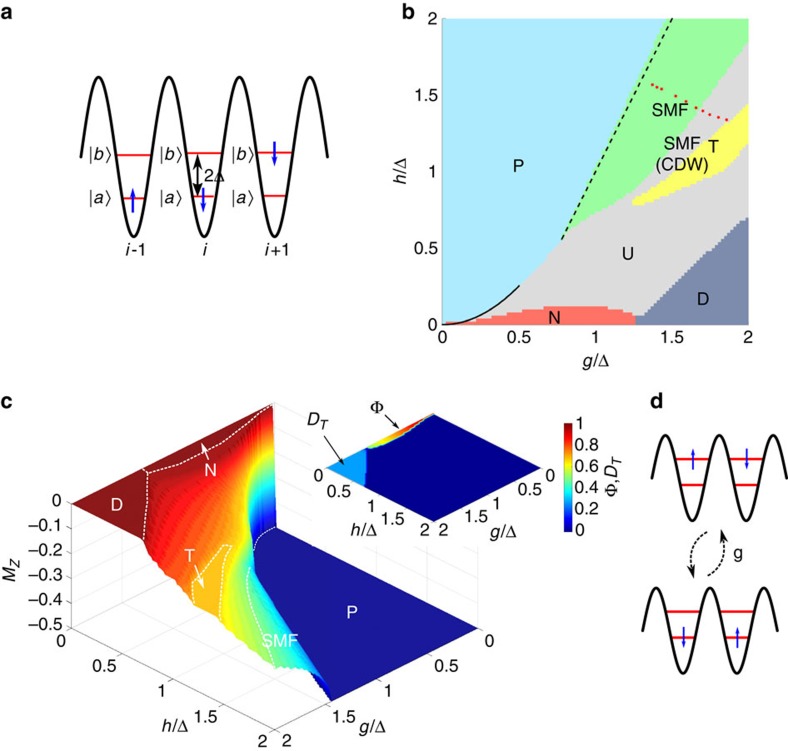
Model and phase diagram for quantum spin-motion coupling. (**a**) Representation of the truncated basis states for over a few sites. The blue arrow indicates the spin, while the two levels represent the motional states 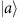 and 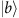, separated by an energy difference 2Δ. (**b**) Ground state phase diagram obtained studying a system of 62 atoms with open boundary conditions with a DMRG algorithm. We identify unambiguously five phases: a paramagnetic phase (P), a Néel ordered phase (N), a dimerized phase of triplets (D), a spin-motion fluid phase (SMF) and a phase of trimers (T). There is an additional phase corresponding to a charge density wave with quasi-long-range order, labeled as SMF(CDW), and whose boundary with a set of still unknown phases U is not well understood. The continuous line is the border of the paramagnetic phase obtained analytically in the weak coupling regime (see text), the dashed line corresponds to *h*=−Δ+2*g*. The 10 red stars indicate parameters (*g*, *h*) where the correlations in Fig. 5c are evaluated. (**c**) Surface plot of the magnetization per atom *M*_*z*_, with the phases of (**b**) indicated. Inset: contour plot of the order parameters |Φ| and |*D*_*T*_|. (**d**) The virtual process (for 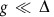) of two atoms exchanging the spin excitation by jumping to the motional state 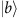 and returning to the original state, which gives rise to the effective Ising interaction term of Hamiltonian (5).

We now consider a quantum description of motion and spins, which is relevant, e.g., if the motion is initially cooled to its ground state. We keep the assumption of tight trapping of the atoms around the minima of the external potential, such that tunneling of atoms between sites can be neglected. We then proceed by projecting the Hamiltonian of equation (2) onto the lowest two motional bands, and denote by 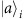 and 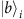 the associated Wannier functions localized around site *i*, as shown in Fig. 3a (see Supplementary Note 3). This represents the minimal model in which spin and motion can couple, since superpositions of states 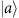 and 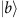 yield spatial wave-functions that are displaced from the site centers. We have also performed calculations involving a third band to verify that the conclusions made from the two-band approximation do not qualitatively change (see Supplementary Note 4 and Supplementary Fig. 2). Within the two-band approximation, it will be convenient to introduce a set of pseudo-spin operators on each site, 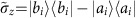, etc., to represent the motional degree of freedom.

The overall Hamiltonian can thus be expressed in terms of these operators as


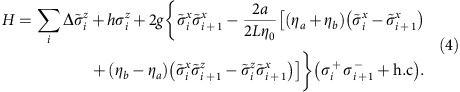


The terms proportional to Δ and *h* describe the energy arising from the band and magnetic field, respectively, while the remainder describes the spin-motion coupling. Here 
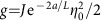
 is a scaled coupling constant, with 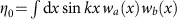 and 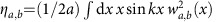 (*w*_*a,b*_ being the Wannier functions of states 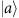 and 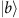). In the following we take *L*=2*a* and the ratio between the trapping lattice depth *V*_L_ and the recoil energy *E*_R_ to be 20, for which numerical evaluation of the Wannier functions yields *η*_0_≈0.54, *η*_*a*_≈0.06 and *η*_*b*_≈0.16. The terms between brackets contain the dependence of the spin interaction on the motional state of the atoms and have a simple physical explanation. The dominant 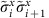 term has largest amplitude when both atoms sit in an equal superposition of states 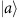 and 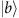 (i.e., the wave-function is maximally displaced from the center), which reflects that the atoms are trapped at nodes of the PCW. The other terms, which are smaller, originate from the exponentially decaying interaction and are responsible for spatial dimerization. It is interesting to note that this Hamiltonian constitutes an extreme case of spin-orbit coupled systems, as neither an orbital kinetic energy nor a motion-independent spin interaction appear.

We study the phase diagram of Hamiltonian (4) in the *g*−*h* plane by means of a finite-size density matrix renormalization group (DMRG) algorithm^35^. The resulting phase diagram for 0≤*g*,*h*≤2Δ is shown in Fig. 3b for *N*=62 atoms, where we can clearly distinguish at least six phases. The procedure by which these phases are numerically demarcated is discussed in detail in Supplementary Note 3, while in the main text we describe the salient physical properties of each phase. First, for sufficiently large magnetic fields *h*>*h*_crit_(*g*), with *h*_crit_(0)=0, the spins are fully polarized and thus the spin-motion coupling has no effect. The many-body state is thus separable, with each atom residing in the lowest motional band, 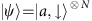 (‘P' phase in Fig. 3b).

Along the *g*-axis up to *g*_crit_ we have a Néel ordered phase ‘N', where the magnetization per atom 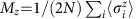 is zero and the Néel order parameter 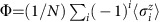 has a finite value, as shown in Fig. 3c. This phase also extends to finite values of *h* with a lobe-like shape. The existence of this phase can be predicted analytically in the weak coupling regime, i.e., for *g*/2Δ small, such that the high-energy excitations associated with populating the upper band can be effectively integrated out. In particular, through a Schrieffer-Wolff transformation^36^ on equation (4) one obtains the following effective Hamiltonian acting only on the spin degrees of freedom:


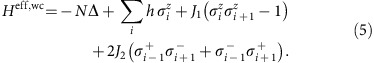


Here *J*_1_=*g*^2^(1+4*χ*^2^)/2Δ, *J*_2_=*g*^2^*χ*^2^/Δ and *χ*=*η*_*a*_/(*η*_0_*L*). Hamiltonian (5) describes a nearest neighbor anti-ferromagnetic (AF) Ising model with an additional XX term coupling next-nearest neighbors, with all such terms mediated by virtual phonons. For example, the spin-motion term in equation (4) proportional to 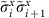 enables a fluctuation where two consecutive atoms, anti-aligned in their spins, jump to the higher band and exchange their spins, before returning to the original state (see Fig. 3d). This process results in a lower energy for the anti-aligned configuration and produces the longitudinal 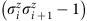 term in (5). For zero magnetic field, given that 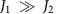 the ground state exhibits AF ordering along *z* (Φ≈1), as illustrated in Fig. 3c. On the other hand, for *h*>*h*_crit_(*g*) all spins are in state 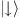. Intuitively, one can expect that the transition from Néel ordering to polarized occurs with all 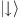 spins in the Néel phase remaining fixed (subchain ‘A'), while the 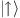 spins (subchain ‘B') ‘melt' and then re-configure pointing downward. One can thus make an ansatz where subchain A acts as an effective magnetic field for B. Thus, subchain B satisfies an XX model with 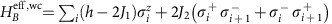, which has two phase transitions to polarized phases (for subchain B) at *h*=2(*J*_1_±*J*_2_). It follows that for *h*<2(*J*_1_−*J*_2_) the total system (A and B) is in the Néel phase, while for *h*>2(*J*_1_+*J*_2_) it is in the P phase. In between the two phases the subchain melts under the effective XX model. Since 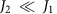, in the *g*−*h* plane this transition region is too narrow to quantitatively match the DMRG results to the XX model predictions, although the effective theory gives correctly the boundary between N and P at *h*_crit_(*g*)≈*g*^2^/Δ for 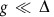 (solid line in Fig. 3b).

**Figure 4:**
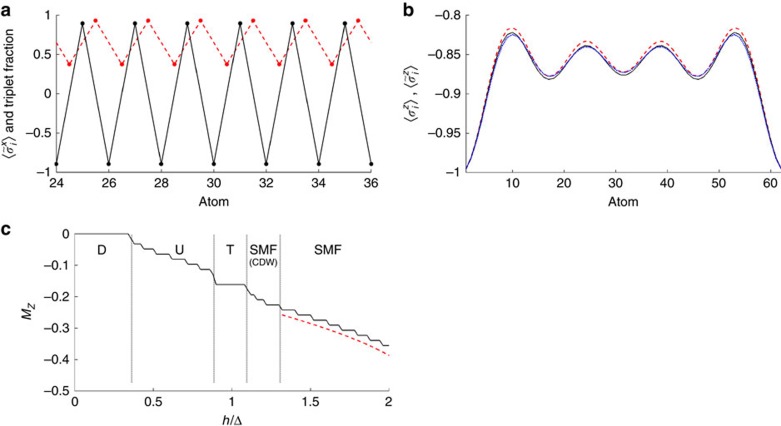
Correlation functions of dimerized and spin-motion fluid phases. (**a**) Spin triplet fraction 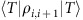 (red dashed line with dots between *i* and *i*+1) and displacement 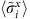 (black solid line) of the ground state for (*g*, *h*)=(1.7, 0.2)Δ, belonging to the dimerized (‘D') phase. Only atoms 24–36 are shown for clarity. (**b**) 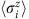 (black solid line), 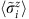 (red dashed line) along the chain for the ground state at (*g*, *h*)=(1.18, 1.4)Δ belonging to the spin-motion fluid (‘SMF') phase. The state contains 4 atoms flipped to 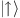 along the direction of the magnetic field. The blue dotted line is 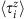 calculated for the ground state of the model Hamiltonian *H*^+^. (**c**) Magnetization curve for *g*=1.6Δ as a function of *h*. The red dashed line is the magnetization predicted by *H*^+^ for the SMF phase.

The Néel order extends to values of 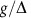
≳1 where the low-energy description of (5) is no longer accurate, and decreases discontinuously to zero with the onset of a new phase of dimerized triplets (labelled ‘D' in Fig. 3b). This phase is characterized by zero magnetization and a non-zero spin triplet dimer order parameter, defined as 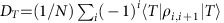 with 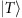 being the spin triplet state and *ρ*_*i*,*i*+1_ the two-site spin reduced density matrix (Fig. 3c). It also has a non-zero spatial dimer order parameter, defined as 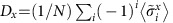. The entangled dimerized structure is evident in Fig. 4a, where we plot the triplet fraction in the two-particle density matrix, 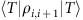 and the displacement 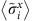 in a part of the chain for (*g*, *h*)=(1.7, 0.2)Δ. Also, we can observe that 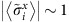. Thus, the two-band approximation for the atomic motion is technically violated since the displacement from the trap center is saturated. However, in Supplementary Note 4, we present calculations involving a third motional band, which allows for a greater maximum displacement of atoms. These calculations exhibit a slower onset of saturation with increasing *g* and no appearance of new phases (at least within the range of parameters considered). Together, this suggests that an exact calculation involving all bands, although directly unfeasible, would produce a result similar to the previously discussed case of classical motion, with a steadily increasing degree of dimerization and triplet fraction with increasing *g*.

For simultaneously large values of *g* and *h*, there is a spin-motion fluid phase (‘SMF') where the system is gapless and the magnetic field strongly polarizes the spins, such that *M*_*z*_ is close to −1/2. This phase corresponds with good approximation to the ground state of the XX Hamiltonian 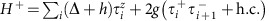. Here 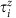 is the Pauli matrix with eigenstates 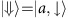 and 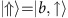, while 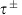 are associated raising and lowering operators. Thus, this phase corresponds to a dilute fluid of composite flips of spin and motion. The existence of this phase can be understood by noting that for large magnetic field, the system is only dilutely populated by spins pointing up. Thus the terms in equation (4) proportional to *η*_*a*,*b*_ that are responsible for dimerization can be neglected. The structure of the remaining Hamiltonian connects naturally the states 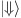 directly to 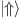, in the form of *H*^+^ (see Supplementary Note 3 for details). The locking between spin and motional correlations can be observed in Fig. 4b, where the expectations values of 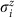 and 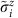 obtained with DMRG are plotted for a representative point in the phase. The oscillations of 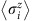 and 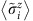 are due to the open boundary conditions in a finite system and are observable also in a pure XX model. In Fig. 4c the magnetization curve predicted by *H*^+^ is compared with the numerical result from the DMRG study of the full Hamiltonian for *g*=1.6Δ, showing good agreement, while in Fig. 3b the predicted boundary with the ‘P' phase *h*_crit_(*g*)≈−Δ+2*g* is represented by a dashed line.

**Figure 5:**
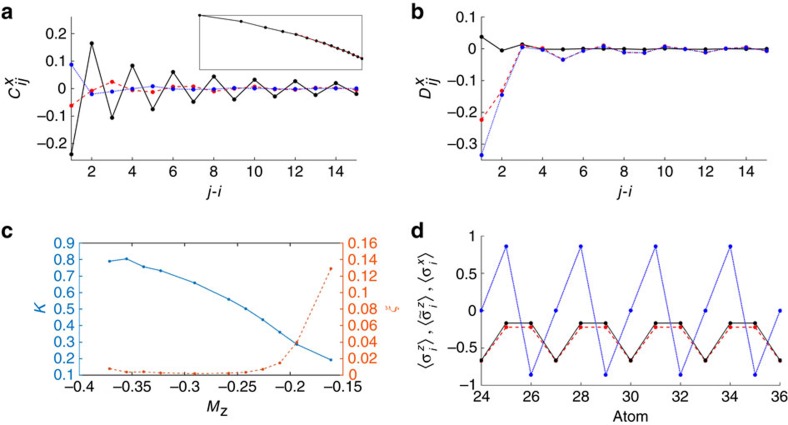
Correlation functions of charge density wave and trimer phases. (**a**) Correlation functions 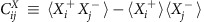 with *X* equal to *τ* (black solid line), 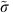 (red dashed line) and *σ* (blue dotted line) at (*g*, *h*)=(1.74, 1.38)Δ, in the SMF(CDW) region of the phase diagram. The value of *i*=29 is taken fixed in the bulk of the chain and *j* is ranging from 30 to 44. Inset: 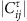 plotted on a log-log scale (black curve), as is the best fit to a Luttinger liquid power-law decay for the points |*i*−*j*|>4 (red dashed line). (**b**) As in (**a**) but for the density correlation functions 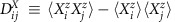. (**c**) In blue: value of the fitted Luttinger parameter *K* as a function of the magnetization, obtained by fitting the long-range part of the 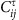 correlation function for the (*g*, *h*) values marked by stars in Fig. 3b. In red: sum of the squares of the residuals *ξ* of the fit. (**d**) 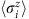 (black solid line), 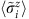 (red dashed line) and 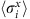 (blue dotted line) along the chain for the ground state at (*g*, *h*)=(1.7, 1.1)Δ, where the ground state belongs to the trimer (‘T') phase.

For 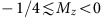, *H*^+^ no longer serves as a good description for the ground state. Most of this region consists of a set of phases ‘U' whose origin is not completely understood yet. However, for strong interactions 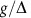
≳1, the system qualitatively appears to behave as an interacting Luttinger liquid for the *τ* particles. Numerical evidence is shown in Fig. 5a, where the two-point correlation functions 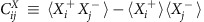 are plotted for various 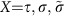, for a representative set of values (*g*, *h*)=(1.74, 1.38)Δ. In particular, if *τ* behaves as a Luttinger liquid, then the long-range decay of interactions is predicted to have a power law form of 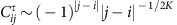 (ref. 37). The inset of Fig. 5a plots the absolute value 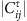 on a log-log scale, which confirms an approximate power law decay. On the other hand, correlations of the other degrees of freedom exhibit more erratic behavior. Similar observations hold for the density correlation functions 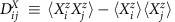 (Fig. 5b). We fit the Luttinger parameter *K* (ref. 37) from the numerical data, taking the ten (*g*, *h*) values indicated by red stars in Fig. 3b across the SMF to U boundary. These fits are performed on the points |*i*−*j*|>4 of 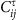, in order to reduce the influence of short-range corrections, which exist even for an ideal Luttinger liquid^37^. The inset of Fig. 5a shows the best fit (red dashed line) for (*g*, *h*)=(1.74, 1.38)Δ, while the fitted values of *K* for all ten chosen (*g*, *h*) points are plotted in Fig. 5c. We have also simultaneously plotted *ξ*, the sum of the squares of the residuals between the best linear fit on a log-log scale and the numerical data. We note that while the choice of region of exclusion of |*i*−*j*|≤4 in taking the fit is somewhat arbitrary, modifying this region (or excluding no points at all) does not change the qualitative conclusions. The decrease below *K*=1 is indicative of the formation of a charge density wave phase with quasi-long-range order, i.e., algebraic decay of the correlation functions. We thus denote this part of the phase diagram as SMF(CDW). The precise boundary of this phase and the nature of the transition to neighboring phases is still not completely understood.

Approaching *M*_*z*_→−1/6 we notice that not only the fitted value of *K* tends to zero but also the quality of the fit decreases rapidly, as indicated by the increase in the residual error *ξ*. This indicates a change of the decay of the correlation function from polynomial to exponential. This is in agreement with the fact that the decrease in *K* is also known to facilitate the possibility of phases with spontaneously broken symmetry, which is observed in our system as well. At *M*_*z*_=−1/6 (one third of the maximum magnetization), we observe indeed the presence of a plateau in the magnetization curve (Figs 3c and 4c), for values of *g* sufficiently large. In this region the ground state assumes a trimerized configuration, as shown in Fig. 5d, where 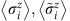 and the displacement 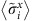 are plotted. While we are not able to predict the appearance of such a plateau in our model from first principles, we note that all of its features are consistent with the conditions of ref. 38. In particular, our Hamiltonian allows for a gapped phase with spontaneously broken symmetry in the ground state with spatial periodicity *n*=3, provided that the quantization condition *n*(*S*−|*M*_*z*_|)=integer is satisfied (here *S*=1/2 is the total spin). Such a gapped phase should be accompanied by a magnetization plateau.

## Discussion

The platform of cold atoms coupled to photonic crystals offers fascinating opportunities to create quantum materials in which spin and motion interact strongly with one another. We have analysed in detail the ground state properties of one experimentally feasible setup, but there exist many exciting avenues for future research. The field of interfacing atoms and photonic crystals is still new and rapidly developing, which makes it difficult to say precisely how the ground state or nearby states can be probed and prepared, but we briefly describe some of the possibilities here. First, it has already been demonstrated that tightly focused optical tweezers can be used to controllably position single atoms nearby nanophotonic structures and couple the atom to the optical mode^39^. Separately, there have been spectacular experiments to create arrays of up to ∼10^2^ atoms in individual optical tweezers^40^,^41^,^42^, and demonstrated capabilities in such systems for motional ground-state cooling and spin readout^40^. An optical tweezer array applied to nanophotonic systems could then be a promising route toward both deterministic positioning of atoms and single-site resolution. Absent single-site measurements, there are a number of global measurements that could be applied to yield signatures of the various phases. For example, it has also been theoretically and experimentally shown^28^,^43^,^44^,^45^ that different atomic spatial patterns can give rise to very different global reflection and transmission spectra for a weak guided probe field. Similar to free space, a guided mode could also be used to efficiently read out global spin properties^46^. In terms of preparation of the ground state, one likely possibility would be through adiabatic evolution (given that the atomic ‘spin' states are internal states that do not readily thermalize). Here, the atoms would be initially optically pumped to a separable state (such as 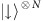), which corresponds to the ground state of a single-particle Hamiltonian *H*^s^. The system could then adiabatically evolve through a Hamiltonian *H*(*t*)=*H*^s^(*t*)+*H*^int^(*t*), where the single-particle Hamiltonian is gradually turned off while the PCW interactions are turned on. Understanding the fidelity of this process requires a more thorough investigation of the excitation spectrum, which itself should exhibit non-trivial properties, including the possibility of signatures of fractional spin^47^.

The strong coupling between spin and motion more broadly invites a number of other intriguing questions. For example, it would be interesting to understand the transport properties when spin and motion strongly hybridize. Moreover, it would be highly interesting to consider models without an external lattice potential, and investigate whether the spin interaction alone can produce full spin-entangled crystallization. One might also consider models where the spin part of the interaction already exhibits non-trivial character, such as frustration or topology. Finally, in terms of applications, it would be interesting to explore whether specially engineered spin-motion Hamiltonians can give rise to useful many-body spin states (such as squeezed states for metrology), when the spin interaction alone is incapable of producing such states.

## Methods

### Density matrix renormalization group

The density matrix renormalization group (DMRG) algorithm is a well-established numerical method for ground state studies of one-dimensional systems^35^. It consists of approximating the exact ground state 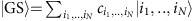 of a finite-size system of *N* sites with local dimension *d* with a matrix product state (MPS), i.e., a state of the form


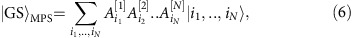


where the matrices *A* have maximum dimension *D*. The DMRG algorithm finds the matrices 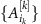 for which 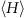 is minimum. This is obtained in an iterative way by optimizing a single matrix in every step, keeping constant all the others and shifting one site at every step. In 2*N* steps the system has been ‘swept' once. The level of approximation can be determined by the relative energy difference d*E* after a sweep. In general for a gapped Hamiltonian, with *D*=100–1000 the energy converges in a few sweeps and one obtains an extremely good approximation of the ground state.

In our numerics we take *D*=100 and the precision on the total energy d*E*=10^−7^ with a maximum number of sweeps equal to 6. For the points in which convergence is not reached within six sweeps *D* is increased to 140 and additional sweeps are performed. We span the *g*−*h* plane with a resolution of 0.02Δ.

### Data availability

The data that support the findings of this study are available from the corresponding author on request.

## Additional information

**How to cite this article:** Manzoni, M. T. *et al*. Designing exotic many-body states of atomic spin and motion in photonic crystals. *Nat. Commun.* **8,** 14696 doi: 10.1038/ncomms14696 (2017).

**Publisher's note**: Springer Nature remains neutral with regard to jurisdictional claims in published maps and institutional affiliations.

## Supplementary Material

Supplementary Information Supplementary Figures, Supplementary Notes and Supplementary References.

## References

[b1] Peierls R. E. Quantum Theory of Solids Clarendon (1955).

[b2] Pytte E. Peierls instability in Heisenberg chains. Phys. Rev. B 10, 4637–4642 (1974).

[b3] Cross M. C. & Fisher D. S. A new theory of the spin-Peierls transition with special relevance to the experiments on TTFCuBDT. Phys. Rev. B 19, 402–419 (1979).

[b4] Bursill R. J., McKenzie R. H. & Hamer C. J. Phase diagram of a Heisenberg spin-Peierls model with quantum phonons. Phys. Rev. Lett. 83, 408–411 (1999).

[b5] Joannopoulos J. D., Johnson S. G., Winn J. N. & Meade R. D. Photonic Crystals: Molding the Flow of Light 2nd edn Princeton University Press (2008).

[b6] John S. & Wang J. Quantum electrodynamics near a photonic band gap: photon bound states and dressed atoms. Phys. Rev. Lett. 64, 2418–2421 (1990). 10041707 10.1103/PhysRevLett.64.2418

[b7] Kurizki G. Two-atom resonant radiative coupling in photonic band structures. Phys. Rev. A 42, 2915–2924 (1990). 9904360 10.1103/physreva.42.2915

[b8] John S. & Wang J. Quantum optics of localized light in a photonic band gap. Phys. Rev. B 43, 12772–12789 (1991). 10.1103/physrevb.43.12772 9997091

[b9] Douglas J. S. . Quantum many-body models with cold atoms coupled to photonic crystals. Nature Photon 9, 326–331 (2015).

[b10] Gonzaléz-Tudela A., Hung C.-L., Chang D. E., Cirac J. I. & Kimble H. J. Subwavelength vacuum lattices and atom-atom interactions in two-dimensional photonic crystals. Nature Photon 9, 320–325 (2015).

[b11] Hung C.-L., Gonzaléz-Tudela A., Cirac J. I. & Kimble H. J. Quantum spin dynamics with pairwise-tunable, long-range interactions. Proc. Natl. Acad. Sci. USA 113, E4946–E4955 (2016). 27496329 10.1073/pnas.1603777113 PMC5003233

[b12] Ni K.-K. . A high phase-space-density gas of polar molecules. Science 322, 231–235 (2008). 18801969 10.1126/science.1163861

[b13] Jin D. & Ye J. Ultracold polar molecules. Many-Body Physics with Ultracold Gases: Lecture Notes of the Les Houches Summer School 94, 273 (2010).

[b14] Micheli A., Brennen G. K. & Zoller P. A toolbox for lattice-spin models with polar molecules. Nature Phys. 2, 341–347 (2008).

[b15] Saffman M., Walker T. G. & Mølmer K. Quantum information with Rydberg atoms. Rev. Mod. Phys. 82, 2313–2363 (2010).

[b16] Porras D. & Cirac J. I. Effective quantum spin systems with trapped ions. Phys. Rev. Lett. 92, 207901 (2004). 15169383 10.1103/PhysRevLett.92.207901

[b17] Richerme P. . Non-local propagation of correlations in quantum systems with long-range interactions. Nature 511, 198–201 (2014). 25008525 10.1038/nature13450

[b18] Jurcevic P. . Quasiparticle engineering and entanglement propagation in a quantum many-body system. Nature 511, 202–205 (2014). 25008526 10.1038/nature13461

[b19] Norcia M. A., Winchester M. N., Cline J. R. K. & Thompson J. K. Superradiance on the milliHertz linewidth strontium clock transition, preprint at https://arxiv.org/abs/1603.05671 (2016). 10.1126/sciadv.1601231 PMC5065256 27757423

[b20] Yu S.-P. . Nanowire photonic crystal waveguides for single-atom trapping and strong light-matter interactions. Appl. Phys. Lett. 104, 111103 (2014).

[b21] Goban A. . Atom-light interactions in photonic crystals. Nature Commun. 5, 3808 (2014). 24806520 10.1038/ncomms4808

[b22] Goban A. . Superradiance for atoms trapped along a photonic crystal waveguide. Phys. Rev. Lett. 115, 063601 (2015). 26296116 10.1103/PhysRevLett.115.063601

[b23] Hood J. D. . Atom-atom interactions around the band edge of a photonic crystal waveguide. Proc. Natl. Acad. Sci. USA 113, 10507 (2016). 27582467 10.1073/pnas.1603788113 PMC5035845

[b24] Lieb E., Schultz T. & Mattis D. Two soluble models of an antiferromagnetic chain. Ann. Phys. 16, 407–466 (1961).

[b25] Gorshkov A. V. . Two-orbital SU(N) magnetism with ultracold alkaline-earth atoms. Nature Phys. 6, 289–295 (2010).

[b26] Taie S. . Realization of a SU(2) × SU(6) system of fermions in a cold atomic gas. Phys. Rev. Lett. 105, 190401 (2010). 21231150 10.1103/PhysRevLett.105.190401

[b27] Trotzky S. . Time-resolved observation and control of superexchange interactions with ultracold atoms in optical lattices. Science 319, 295–299 (2008). 18096767 10.1126/science.1150841

[b28] Chang D. E., Cirac J. I. & Kimble H. J. Self-organization of atoms along a nanophotonic waveguide. Phys. Rev. Lett. 110, 113606 (2013). 25166535 10.1103/PhysRevLett.110.113606

[b29] Baumann K., Guerlin C., Brennecke F. & Esslinger T. Dicke quantum phase transition with a superfluid gas in an optical cavity. Nature 464, 1301–1306 (2010). 20428162 10.1038/nature09009

[b30] Klinder J., Keßler H., Reza Bakhtiari M., Thorwart M. & Hemmerich A. Observation of a superradiant Mott insulator in the Dicke-Hubbard model. Phys. Rev. Lett. 115, 230403 (2015). 26684102 10.1103/PhysRevLett.115.230403

[b31] Black A. T., Chan H. W. & Vuletić V. Observation of collective friction forces due to spatial self-organization of atoms: from Rayleigh to Bragg scattering. Phys. Rev. Lett. 91, 203001 (2003). 14683358 10.1103/PhysRevLett.91.203001

[b32] Asbóth J. K., Domokos P., Ritsch H. & Vukics A. Self-organization of atoms in a cavity field: threshold, bistability, and scaling laws. Phys. Rev. A 72, 053417 (2005).

[b33] Domokos P. & Ritsch H. Collective cooling and self-organization of atoms in a cavity. Phys. Rev. Lett. 89, 253003 (2002). 12484881 10.1103/PhysRevLett.89.253003

[b34] Jordan P. & Wigner E. Über das Paulische Äquivalenzverbot. Z. Physik 47, 631–651 (1928).

[b35] Schollwöck U. The density-matrix renormalization group in the age of matrix product states. Ann. Phys. 326, 96–192 (2011).

[b36] Bravyi S., DiVincenzo D. P. & Loss D. Schrieffer-Wolff transformation for quantum many-body systems. Ann. Phys. 326, 2793–2826 (2011).

[b37] Giamarchi T. Quantum physics in one dimension Clarendon (2004).

[b38] Oshikawa M., Yamanaka M. & Affleck I. Magnetization plateaus in spin chains: ‘Haldane gap' for half-integer spins. Phys. Rev. Lett. 78, 1984–1987 (1997).

[b39] Tiecke A. V. . Nanophotonic quantum phase switch with a single atom. Nature 508, 241–244 (2014). 24717513 10.1038/nature13188

[b40] Kaufman A. M. . Two-particle quantum interference in tunnel-coupled optical tweezers. Science 345, 306–309 (2014). 24968938 10.1126/science.1250057

[b41] Barredo D., de Léséleuc S., Lienhard V., Lahaye T. & Browaeys A. An atom-by-atom assembler of defect-free arbitrary 2D atomic arrays. Preprint at https://arxiv.org/abs/1607.03042 (2016). 10.1126/science.aah3778 27811285

[b42] Endres M. . Cold matter assembled atom-by-atom. Preprint at https://arxiv.org/abs/1607.03044 (2016).

[b43] Chang D. E., Jiang L., Gorshkov A. V. & Kimble H. J. Cavity QED with atomic mirrors. New. J. Phys. 14, 063003 (2012).

[b44] Corzo N. V. . Large Bragg reflection from one-dimensional chains of trapped atoms near a nanoscale waveguide. Phys. Rev. Lett. 117, 133603 (2016). 27715121 10.1103/PhysRevLett.117.133603

[b45] Sørensen H. L. . Coherent backscattering of light off one-dimensional atomic strings. Phys. Rev. Lett. 117, 133604 (2016). 27715084 10.1103/PhysRevLett.117.133604

[b46] Hammerer K., Sørensen A. S. & Polzik E. S. Quantum interface between light and atomic ensembles. Rev. Mod. Phys. 82, 1041–1093 (2010).

[b47] Okunishi K. & Tonegawa T. Fractional *S*^*z*^ excitation and its bound state around the 1/3 plateau of the *S*=1/2 Ising-like zigzag XXZ chain. Phys. Rev. B 68, 224422 (2003).

