# Supplementary material for: Designing exotic many-body states of atomic spin and motion in photonic crystals
Source: Nat Commun. 2017 Mar 8;8:14696. doi: 10.1038/ncomms14696 (PMC5344972; doi:10.1038/ncomms14696)
Supplement: Supplementary Information — Supplementary Figures, Supplementary Notes and Supplementary References. [file ncomms14696-s1.pdf]

Supplementary note 1. Derivation of Hamiltonian

Here, we present a more detailed derivation of Eq. (1) in the main text, which describes how an ensemble of atoms interacts via the process of photon exchange near a photonic crystal. We generally begin by considering an ensemble of two-level atoms with ground state $|g\rangle$ and excited state $|e\rangle$, with corresponding transition frequency ω_{eg}. The atoms can be in the vicinity of any linear, isotropic dielectric materials, characterized by dimensionless electric permittivity $\epsilon(r,\omega)$. Here r denotes the spatial coordinate, and ϵ in general is allowed to be dependent on frequency ω and absorbing (i.e. have an imaginary component). A quantum theory of atom-light interactions in the presence of such dielectric media has been pioneered in a number of works by Welsch and co-workers [1, 2]. Here we will not go through the derivation again, but will present the main results and qualitatively argue why the results are physically reasonable.

Intuitively, two-level atoms can interact via the electromagnetic field through the exchange of photons. Microscopically, one atom would be able to de-excite by emitting a photon (as characterized by the atomic lowering operator $\sigma^{ge} \equiv |g\rangle\langle e|$ and another atom would be able to absorb the photon and become excited (as characterized by the raising operator $\sigma^{eg} \equiv |e\rangle\langle g|$). By integrating out the field, one obtains an effective atom-atom interaction Hamiltonian of the form [2]

$$H = -\mu_0\omega_{eg}^2 \sum_{i,j} \wp^* \cdot (\mathrm{Re}\,\mathbf{G}(r_i, r_j, \omega_{eg})) \cdot \wp\, \sigma_i^{eg}\sigma_j^{ge}. \tag{1}$$

Here $\wp$ is the dipole matrix element of the transition, and $\mathbf{G}$ is the classical electromagnetic Green's function, defined as the solution to the wave equation with a point source,

$$\left[(\nabla\times\nabla\times) - \omega^2\epsilon(r,\omega)/c^2\right]\mathbf{G}(r,r',\omega) = \delta(r-r')\otimes I. \tag{2}$$

The Green's function $\mathbf{G}$ is in fact a 3x3 matrix, whose elements G_{ab} have the meaning of being the field at r projected along a ($a = x, y, z$), due to an oscillating source of frequency ω at r', whose dipole moment is oriented along b. For simplicity, from here forward we will not explicitly indicate the tensor nature of $\mathbf{G}$, *e.g.*, by considering a transition that is linearly polarized along x, $\wp \propto \hat{x}$, such that only the G_{xx} component is relevant (and thus dropping the subscripts). Physically, although a two-level system produces non-classical light, classical and quantum fields *propagate* the same way, and thus the coherent interaction strength between the atoms can be characterized by the classical Green's function. Moreover, the dependence of H on the real part of G has a classical analogy, in that a field in phase with an oscillating dipole stores time-averaged energy.

Likewise, an ensemble of atoms should experience dissipation in the form of spontaneous emission. Eliminating the fields results in a corresponding master equation for the density matrix ρ of the atoms alone, with Lindblad operator given by

$$L[\rho] = \sum_{ij} \frac{\mu_0\omega_{eg}^2}{\hbar}\, \wp^* \cdot (\mathrm{Im}\,\mathbf{G}(r_i, r_j, \omega_{eg})) \cdot \wp\, \left(2\sigma_i^{ge}\rho\sigma_j^{eg} - \sigma_i^{eg}\sigma_j^{ge}\rho - \rho\sigma_i^{eg}\sigma_j^{ge}\right), \tag{3}$$

where we have expressed explicitly the tensor nature of the Green's function. This equation also has a classical analogy, in that the field out of phase with an oscillating dipole performs time-averaged work. As a simple limit, one can consider the case of a single atom in vacuum, for which $\epsilon(r,\omega) = 1$. The corresponding Green's function G_0 has the property that $\mathrm{Im}\,G(r,r,\omega_{eg}) = \omega_{eg}/(6\pi c)$. Substituting this into $L[\rho]$ one finds that $L[\rho] = \Gamma_0(2\sigma^{ge}\rho\sigma^{eg} - \sigma^{ee}\rho - \rho\sigma^{ee})$, where $\Gamma_0 = \omega_{eg}^3\wp^2/(3\pi\epsilon_0\hbar c^3)$ correctly identifies as the single-atom free-space spontaneous emission rate.

Supplementary Equations (1) and (3) are quite general and completely dictate the atomic dynamics given knowledge of the Green's function G. For an actual photonic crystal structure such as the "alligator" PCW used in experiments [3, 4], the Green's function G can be numerically calculated using standard electromagnetic simulation software, as has been done in Ref. [5]. G contains information about fundamental dissipation rates such as atomic spontaneous emission into free space, and in principle numerical simulations could also incorporate any kind of imperfections stemming from structure disorder (that can be captured in some imperfect dielectric profile $\epsilon(r,\omega)$) to infer its effect on atom-atom interactions. However, in Ref. [5] it was shown that the predictions from numerical simulations of G for a realistic photonic crystal waveguide agree quantitatively with a simpler theoretical model of atom-atom interactions at a band edge. We thus present the simple model below, which provides excellent intuition about the strengths of the coherent interactions and dissipation, and the effect of certain imperfections.

In particular, we consider an idealized 1D model of atoms interacting via a band edge of a photonic crystal structure. The two-level atoms are assumed to predominantly couple to a single band, whose dispersion relation can be expanded quadratically around the band edge, $\omega(q) = \omega_{\mathrm{b}}(1 - \alpha(q-k)^2/k^2)$ (see Supplementary Figure 1a). Here, ω_{b} is the frequency at the band edge, q is the Bloch wavevector of the guided mode, $k = \pi/a$ is the edge of the Brillouin zone

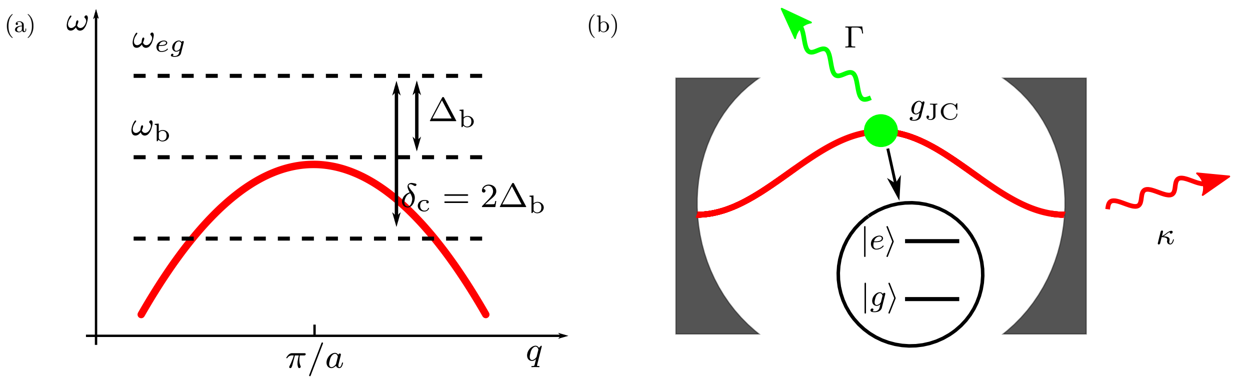

Supplementary Figure 1: **Atom interactions at the band edge.** (a) Energy level structure for a two-level atom coupled to a photonic crystal. The transition frequency ω_{eg} has a detuning $\Delta_{\rm b}$ above the band-edge frequency $\omega_{\rm b}$. $\delta_{\rm c} = 2\Delta_{\rm b}$ is the detuning of the atom from the effective "cavity" mode frequency. (b) Schematic representation of the Jaynes-Cummings model: a two-level atom is coupled to a cavity mode with coupling strength $g_{\rm JC}$. The atom can decay to free space at a rate Γ (green arrow), while the cavity mode decays at a rate κ (red arrow).

determined by the structure periodicity a, and $\alpha > 0$ is a dimensionless parameter characterizing the band curvature. The atomic transition frequency $\omega_{eg} > \omega_{\rm b}$ is assumed to lie within the band gap and couple to an upper band edge. The conclusions below would also hold if the atoms were coupled to a lower band edge, but with a change in the sign of the resulting atom-atom interaction.

The Hamiltonian describing the coupled atom-photonic crystal system is given by $H = H_0 + V$, where

$$H_0 = \sum_j \omega_{eg}\sigma_j^{ee} + \int dq\, \omega(q) a_q^\dagger a_q, \tag{4}$$

$$V = g\sum_j \int dq (\sigma_j^{eg} a_q u_q(x_j) e^{\mathrm{i}qx_j} + \mathrm{h.c.}). \tag{5}$$

Here a_q is the annihilation operator associated with guided mode q, and $u_q(x)$ is a dimensionless periodic Bloch function associated with the electric field profile of the guided mode. The interaction strength g is given by $g = \wp\sqrt{\omega_{\rm b}/(4\pi\epsilon_0\hbar A)}$, where A is the effective mode cross-sectional area.

Before deriving the atom-atom interactions, we first note that while the dispersion relation $\omega(q)$ provides the frequency of the guided modes q, it also has physical meaning for frequencies in the band gap. In particular, defining $\Delta_{\rm b} = \omega_{eg} - \omega_b > 0$ as the detuning of the atomic frequency from the band edge and substituting it in for $\omega(q)$, one finds an imaginary wavevector q as the solution, $q - k = \mathrm{i}\sqrt{k^2\Delta_{\rm b}/\alpha\omega_{\rm b}}$. This describes an evanescently decaying field, with a corresponding attenuation length of $L = 1/\mathrm{Im}\,(q-k) = \sqrt{\alpha\omega_{\rm b}/k^2\Delta_{\rm b}}$. This is the length over which the field from a dipole source would attenuate if its frequency were within the band gap. We now proceed to eliminate the photonic modes to arrive at an effective atom-atom interaction. Beginning with the manifold of states consisting of any number of atomic ground and excited states and zero photons, $\{|g\rangle, |e\rangle\}^{\otimes N} \otimes |0\rangle$, the interaction Hamiltonian V couples these states to a manifold with one fewer excited atom and one photon in mode q, $|1_q\rangle$. The goal is to treat the fluctuations to the manifold containing one photon within second-order perturbation theory and project the effective system dynamics back to the zero-photon manifold (*e.g.*, by Schrieffer-Wolff transformation), resulting in a purely atomic interaction. The derived effective Hamiltonian takes the form [5]

$$H^{\rm eff} = \frac{g_{\rm c}^2}{2\Delta_{\rm b}} \sum_{ij} u_k(x_i)u_k(x_j) \exp\left(-|x_i - x_j|/L\right) \sigma_i^{eg}\sigma_j^{ge}, \tag{6}$$

where $g_c = \sqrt{2\pi/L}g$ (note that g is a coupling strength to a continuum and has units $\mathrm{s}^{-1}\sqrt{\mathrm{m}}$, so g_c has units of s^{-1}). Here, we have assumed that the spatial Bloch modes $u_q \approx u_k$ can be treated as nearly constant near the band edge. For realistic PhC structures they appear sinusoidal $u_k(x) = \cos\pi x/a$ along the axis of the waveguide, *i.e.* at the band edge, the modes form a standing wave exactly as in a Fabry-Perot cavity. The mode area A (which enters in g) and the band curvature α can be calculated independently from numerical simulations for a realistic structure, and

upon doing so one finds that the simple model of Supplementary Equation (6) quantitatively agrees with full Green's function simulations without any free fitting parameters [5].

Writing the effective Hamiltonian in the form of Supplementary Equation (6) in fact suggests an elegant interpretation. Aside from the exponential spatial dependence $e^{-|x_i-x_j|/L}$, the interaction is exactly what one would find for atoms coupled to an off-resonant cavity within the Jaynes-Cummings model [5–7] (see Supplementary Figure 1b). In particular, the Jaynes-Cummings Hamiltonian is given by

$$H^{\rm JC} = \delta_{\rm c} \sum_i \sigma_i^{ee} + g_{\rm JC} \sum_i \cos kx_i (\sigma_i^{eg} a + {\rm h.c.}), \tag{7}$$

where $g_{\rm JC}$ is the single-atom vacuum Rabi splitting of the cavity, a is the annihilation operator of the cavity mode, and $\delta_{\rm c}$ is the atom-cavity detuning. In the far-detuned regime $|\delta_{\rm c}| > g_{\rm JC}$ the off-resonant photons can be eliminated to yield an effective atom-atom Hamiltonian $H^{\rm JC,eff} = (g_{\rm JC}^2/\delta_{\rm c}) \sum_{ij} \cos kx_i \cos kx_j \sigma_i^{eg} \sigma_j^{ge}$. Compared to Supplementary Equation (6), this suggests that the PhC interaction can be understood as arising from an effective cavity, with the mapping $g_{\rm JC} = g_{\rm c}$ and $\delta_{\rm c} = 2\Delta_{\rm b}$ (*i.e.* the "cavity" mode for the PhC sits $\Delta_{\rm b}$ below the band edge, see Supplementary Figure 1a). The photon associated with this "cavity" mode is simply that exponentially localized around an excited atom, unable to propagate in the waveguide due to the band gap.

This analogy can in fact be made more formal [5]. In particular, in a real cavity the vacuum Rabi splitting scales with mode volume as $g_{\rm JC} \propto 1/\sqrt{V}$, and it can be shown that the interaction strength $g_{\rm c} \propto 1/\sqrt{AL}$ in the PhC is exactly the same as a real cavity of the same size. Within the Jaynes-Cummings model, the role of losses is well understood, and one can exploit this mapping to predict the effect of dissipation in the PhC. Specifically, within the Jaynes-Cummings model, two fundamental dissipation channels are the spontaneous emission rate of an excited state atom into free space (at a rate Γ typically comparable to the vacuum emission rate Γ_0), and the decay of the cavity photon at a rate κ (see Supplementary Figure 1b). To present a concrete example of the influence of dissipation within the Jaynes-Cummings model, we consider the case of two atoms, and investigate the optimal fidelity in which they can coherently exchange a spin excitation (assuming each atom is trapped at an antinode, $\cos kx_i = 1$). In particular, we begin with one atom initially in the excited state while the other is in the ground state, $|\psi(t=0)\rangle = |eg\rangle$. As the coherent exchange rate from Supplementary Equation (6) is $g_{\rm JC}^2/\delta_{\rm c}$, one must wait a time $\tau \approx \pi\delta_{\rm c}/2g_{\rm JC}^2$ in order for the state to oscillate to $|\psi(\tau)\rangle \approx |ge\rangle$. On the other hand, during that time, the accumulated loss probability is given by $\mathcal{L} \approx \tau(\Gamma + \kappa(g_{\rm JC}/\delta_{\rm c})^2)$, where $(g_{\rm JC}/\delta_{\rm c})^2$ represents the fraction of population by which the atomic excited state is dressed by a cavity photon (and thus picks up the corresponding photon losses). Minimizing $\mathcal{L}$ with respect to δ_c, one finds that $\mathcal{L}_{\rm min} \approx \pi/\sqrt{C}$, where $C = g_{\rm JC}^2/(\kappa\Gamma)$ is the single-atom cooperativity factor. Thus, the strength of coherent interactions (when optimized) can exceed that of dissipation by an amount $\sim \sqrt{C}$. C can be re-written as $C \sim Q\lambda^3/V$, which depends on the quality factor $Q = \omega/\kappa$ of the cavity and its mode volume.

In a PhC, the same loss mechanisms occur. An atom trapped near a PhC emits into free space at a rate $\sim \Gamma_0$, and the photon localized around an excited atom sees the absorption and scattering imperfections of the dielectric to decay at a rate κ. This rate κ should be similar to PhC cavities made from the same material and fabrication processes, for which quality factors of $Q > 10^5$ have been achieved. Assuming an interaction length of $L \sim \lambda$, this translates into an effective vacuum Rabi splitting of $g_{\rm c}/(2\pi) \sim 10$ GHz (for a Cs transition) and a cooperativity of $C_\lambda \sim 10^4$.

In addition to the fundamental dissipation mechanisms of photon loss and atomic spontaneous emission analyzed above, one can envision a number of technical imperfections as well. This includes the possible role of surface charge, adsorbed atoms, etc. Many of these are difficult to theoretically predict and must wait for more careful experimental characterization. Promisingly, while there have not been any precision measurements, existing experiments and their spectra seem to be well-captured by the models of Supplementary Equations (1), (3), and (6), indicating that the effect of other imperfections lies below the characteristic scale of photon-mediated atom-atom interactions. One additional imperfection, which is possible to theoretically analyze, is local disorder in the waveguide, which results in Anderson localization for otherwise freely propagating photons in the band. In unpublished work, we have analyzed the effect of Anderson localization on the statistics of atom-atom interaction strengths. We have found that when the Anderson localization length ξ exceeds the predicted interaction length L of the clean system, then the variance in interaction strength is negligible (where the averaging is done over many random configurations of local disorder). On the other hand, when $\xi < L$, the properties of the atom-atom interaction become highly random and no longer reflect the predictions of the clean system.

Having explained on general grounds the possible achievable strengths of interactions in photonic crystals and the relevant dissipation mechanisms, we now apply these results specifically to the case of observing spin-motion coupling. First, it should be noted that while the photon-mediated interactions in PhC's occur via the excited state, it is generally not convenient to directly work with excited states. In particular, we want to observe the influence of spin-motion coupling in competition with an external trapping potential for the atoms. Typical external trap

frequencies are below $\omega_{\rm m}/2\pi \lesssim 1$ MHz, which is much smaller than the achievable bare interaction strengths in PhC structures (*e.g.*, $g_{\rm c}/(2\pi) \sim 10$ GHz), and the excited state decay rate ($\Gamma_0 \sim 2\pi \times 5$ MHz for Cs). Ideally one would like the interaction strength to be comparable to trapping energies, while making dissipation much smaller. To achieve this, one can work within a hyperfine ground-state manifold, employing an additional state $|s\rangle$ within the manifold as illustrated in Fig. 1a of the main text (here the states $|g\rangle$ and $|s\rangle$ are represented by the "spin" states $|\downarrow\rangle, |\uparrow\rangle$, respectively). A classical control beam Ω_L facilitates Raman transitions between $|g\rangle, |s\rangle$ via the excited state $|e\rangle$. For a control beam detuning Δ_L, the excited state $|e\rangle$ can be eliminated [5], resulting in a Hamiltonian identical in form to Supplementary Equation (6),

$$H^{\rm int} = \frac{g_{\rm c}^2}{2\Delta_{\rm b}} \left(\frac{\Omega_{\rm L}}{\Delta_{\rm L}}\right)^2 \sum_{ij} u_k(x_i) u_k(x_j) \exp\left(-|x_i - x_j|/L\right) \sigma_i^{sg} \sigma_j^{gs}, \tag{8}$$

but with state $|e\rangle$ replaced with state $|s\rangle$ and the interaction strength reduced by a factor $(\Omega_{\rm L}/\Delta_{\rm L})^2$. This is identical to our starting Hamiltonian, Eq. (1), in the main text, where $J = g_{\rm c}^2 \Omega_{\rm L}^2/(2\Delta_{\rm b}\Delta_{\rm L})$ is a single parameter capturing all of the details of the interaction.

Using a Raman process also decreases the optimized dissipation rates by the same factor of $(\Omega_{\rm L}/\Delta_{\rm L})^2$, such that the square root of cooperativity $\sqrt{C}$ still describes the ratio between the rates of coherent interactions and dissipation [5]. Thus, the general strategy to observe coherent spin-motion coupling is to choose $(\Omega_{\rm L}/\Delta_{\rm L})^2$ such that J becomes comparable to the energy scale of external trapping (*e.g.*, $V_{\rm L}$ in Eq. (2) of the main text), which ensures that dissipation is highly suppressed compared to the energy scales in the ideal Hamiltonian. It should also be noted that the Raman process enables the Hamiltonian $H^{\rm int}(t)$ to become time-dependent, if the control field amplitude $\Omega_{\rm L}(t)$ is varied in time.

Once such experimental conditions are in place, there is an additional question of how a ground state in some part of the phase diagram (Fig. 3b of main text) can be prepared, particularly since the "spins" are atomic internal states that are not connected with any thermal bath. One possibility is via adiabatic preparation. In particular, the atoms could be optically pumped into an initial separable state (such as $|g\rangle^{\otimes N}$), the ground state of a single-particle Hamiltonian $H^{\rm s}$. The system can then be adiabatically evolved in time, $H(t) = H^{\rm s}(t) + H^{\rm int}(t)$, with $H^{\rm s}$ turned off while the PhC interaction is gradually turned on. The fidelity of remaining in the ground state through such a process is an interesting but open question, which will be dealt with in further work. In particular, it requires a better understanding of the low-lying excitation spectrum, and the implementation of time-dependent simulations (such as time-dependent DMRG). It is also possible that once $H^{\rm int}$ is experimentally realized, interesting phenomena can be observed directly in non-equilibrium dynamics.

Supplementary note 2. Classical motion

Hamiltonian (2) of the main text, in the nearest-neighbor approximation and under the ansatz

$$\delta_i = (-1)^i \delta \tag{9}$$

on the atomic positions, becomes

$$H(\delta) = \frac{NV_{\rm L}}{2} \sin^2 k\delta/2 - J \sum_i \sin^2 k\delta \, \exp[-2(a + (-1)^i \delta)/L] \, (\sigma_i^+ \sigma_{i+1}^- + {\rm h.c}) + h\, \sigma_i^z. \tag{10}$$

The spin operators can be mapped to fermion annihilation and creation operators $c_i, c_i^\dagger$ using the using Jordan-Wigner transformation [8]

$$\sigma_i^+ = c_i^\dagger \, e^{{\rm i}\pi \sum_{l<i} c_l^\dagger c_l}, \tag{11}$$

$$\sigma_i^- = e^{-{\rm i}\pi \sum_{l<i} c_l^\dagger c_l} \, c_i, \tag{12}$$

$$\sigma_i^z = 2c_i^\dagger c_i - 1, \tag{13}$$

which transforms Supplementary Equation (10) into

$$H(\delta) = E^{\rm tr}(\delta) - J \sum_i \sin^2 k\delta \, \exp[-2(a + (-1)^i \delta)/L] \, (c_i^\dagger c_{i+1} + {\rm h.c}) + h\,(2c_i^\dagger c_i - 1), \tag{14}$$

where $E^{\rm tr}(\delta) = NV_{\rm L}/2\,\sin^2 k\delta/2$. We note that, because of the dimerization ansatz, two different couplings appear now in the Hamiltonian:

$$J_{\rm S}(\delta) \;=\; J\,\sin^2 k\delta\,\exp[-2(a-\delta)/L], \tag{15}$$
$$J_{\rm W}(\delta) \;=\; J\,\sin^2 k\delta\,\exp[-2(a+\delta)/L]. \tag{16}$$

Furthermore, it is natural to relabel the atoms by dimerized pairs (indexed by "j") and the position of the atom inside the pair, i.e. left or right. Then Hamiltonian of Supplementary Equation (10) can be expressed as

$$H(\delta) = E^{\rm tr}(\delta) - J_{\rm S}(\delta)\sum_j (c^\dagger_{j,L}c_{j,R} + {\rm h.c}) - J_{\rm W}(\delta)\sum_j (c^\dagger_{j,R}c_{j+1,L} + {\rm h.c}) + 2h\sum_j (c^\dagger_{j,L}c_{j,L} + c^\dagger_{j,R}c_{j,R} - 1). \tag{17}$$

In this way we have divided the interactions between atoms in the same dimer and atoms in neighboring dimers.

We perform now a change of basis in each dimer, introducing the fermionic operators

$$s_j \;=\; \frac{1}{\sqrt{2}}(c_{L,j} - c_{R,j}), \tag{18}$$
$$t_j \;=\; \frac{1}{\sqrt{2}}(c_{L,j} + c_{R,j}) \tag{19}$$

in terms of which Supplementary Equation (17) is

$$H(\delta) = E^{\rm tr}(\delta) - J_{\rm S}(\delta)\sum_j (t^\dagger_j t_j - s^\dagger_j s_j) - \frac{J_{\rm W}(\delta)}{2}\sum_j (t^\dagger_j t_{j+1} - s^\dagger_j s_{j+1} + t^\dagger_j s_{j+1} - s^\dagger_j t_{j+1} + {\rm h.c}) + \\ + 2h\sum_j (t^\dagger_j t_j + s^\dagger_j s_j - 1). \tag{20}$$

In Fourier space, Supplementary Equation (20) becomes

$$H(\delta) = E^{\rm tr}(\delta) - Nh - \sum_q \begin{pmatrix} t^\dagger_q & s^\dagger_q \end{pmatrix} \begin{pmatrix} J_{\rm S}(\delta) + J_{\rm W}(\delta)\,\cos q - 2h & +{\rm i}J_{\rm W}(\delta)\,\sin q \\ -{\rm i}J_{\rm W}(\delta)\,\sin q & -J_{\rm S}(\delta) - J_{\rm W}(\delta)\,\cos q - 2h \end{pmatrix} \begin{pmatrix} t_q \\ s_q \end{pmatrix}, \tag{21}$$

which can be easily diagonalized as

$$H(\delta) = E^{\rm tr}(\delta) - Nh + \sum_q \Big[(2h + \epsilon_q)\, d^\dagger_q d_q + (2h - \epsilon_q)\, u^\dagger_q u_q\Big]. \tag{22}$$

The spectrum is given by

$$\epsilon_q(\delta) = \Big(J^2_{\rm S}(\delta) + J^2_{\rm W}(\delta) + 2J_{\rm S}(\delta)J_{\rm W}(\delta)\cos q\Big)^{1/2} = J\,e^{-2a/L}\,\sin^2 k\delta\,\Big(4\cosh^2 2\delta/L + 2(\cos q - 1)\Big)^{1/2} \tag{23}$$

and

$$d_q \;=\; \frac{1}{\sqrt{(\epsilon_q + a_q)^2 + b_q^2}}\Big({\rm i}(\epsilon_q + a_q)t_q + b_q s_q\Big), \tag{24}$$
$$u_q \;=\; \frac{1}{\sqrt{(\epsilon_q - a_q)^2 + b_q^2}}\Big(-{\rm i}(\epsilon_q - a_q)t_q + b_q s_q\Big), \tag{25}$$

with $a_q = J_{\rm S}(\delta) + J_{\rm W}(\delta)\,\cos q$ and $b_q = J_{\rm W}(\delta)\,\sin q$.

Since ϵ_q is positive for every q and J has been assumed to be positive, the ground state involves only u operators and is equal to

$$|{\rm GS}\rangle_\delta = \Big(\prod_{q|\epsilon_q(\delta)>2h} u^\dagger_q\Big)\,|0\rangle\,. \tag{26}$$

The ground state energy per atom is:

$$E(\delta) \; = \; E^{\rm tr}(\delta) - h - \frac{1}{N} \sum_{q|\epsilon_q(\delta)>2h} 2h - \epsilon_q(\delta). \tag{27}$$

The interaction energy per atom (i.e., the term proportional to J) has an analytical expression for $h=0$, given by

$$E^{\rm int,h=0}(\delta) = -\frac{2J\,e^{-2a/L}}{\pi}\,\sin^2 k\delta\,\cosh 2\delta/L\,E(\cosh^{-1} 2\delta/L). \tag{28}$$

Here we have taken the thermodynamic limit and replaced the summation on q by an integral and $E(k)$ denotes the complete elliptic integral of the second kind.

From Supplementary Equation (26) we can calculate the triplet (and singlet) fraction for two atoms within a dimer, which is defined as

$$T_{\rm S}(\delta) = \frac{1}{N}\sum_j \langle t_j^\dagger t_j(1 - s_j^\dagger s_j)\rangle = \frac{1}{N}\sum_j \langle t_j^\dagger t_j\rangle - \langle s_j^\dagger s_j t_j^\dagger t_j\rangle\,, \tag{29}$$

where the expectation value is taken on the ground state. For $h=0$ we can calculate it analytically. Inverting Supplementary Equation (24) and using the expression for t and s as function of u and d we find that

$$T_{\rm S}^{h=0}(\delta) = \left(\frac{1}{2} + I_{\rm S}(\delta)\right)^2, \tag{30}$$

where

$$I_{\rm S}(\delta) = \frac{1}{2\pi}\int_{-\pi}^{\pi} dq\,\frac{a_q}{2\epsilon_q}. \tag{31}$$

$I_{\rm S}(\delta)$ tends to $1/\pi$ for $\delta \to 0$. Similarly we can calculate the triplet fraction for consecutive atoms in different dimers

$$T_{\rm W}(\delta) = \frac{1}{N}\sum_j \langle t_j'^\dagger t_j'(1 - s_j'^\dagger s_j')\rangle = \frac{1}{N}\sum_j \langle t_j'^\dagger t_j'\rangle - \langle s_j'^\dagger s_j' t_j'^\dagger t_j'\rangle\,, \tag{32}$$

with $t_j' = 1/2(t_j - s_j + t_{j+1} + s_{j+1})$ and $s_j' = 1/2(t_j - s_j - t_{j+1} - s_{j+1})$, which for $h=0$ is equal to

$$T_{\rm W}^{h=0}(\delta) = \left(\frac{1}{2} + I_{\rm W}(\delta)\right)^2, \tag{33}$$

where

$$I_{\rm W}(\delta) = \frac{1}{2\pi}\int_{-\pi}^{\pi} dq\,\frac{a_q\cos q + b_q \sin q}{2\epsilon_q}. \tag{34}$$

$I_{\rm W}(\delta)$ tends to $1/\pi$ for $\delta \to 0$.

Supplementary note 3. Two-band model of quantum motion

We begin by deriving the Hamiltonian given by Eq. (4) in the main text, describing spin-motion coupling in the two-band approximation. We assume that the atoms are trapped tightly enough in the minima of the trapping potential that the overlap of the wavefunctions of atoms at neighboring sites is negligible. As a consequence we can neglect tunneling of atoms between sites and quantum statistics. Furthermore we assume that only the ground and first excited Wannier motional states $|a\rangle_i$ and $|b\rangle_i$ associated with each site are relevant. We check *a posteriori* the validity of this two-band approximation by comparing with numerical results including the second excited state, as discussed further below.

Projecting the interaction term $J\,e^{-|x-x'|/L}\,\sin kx \sin kx'\,(\sigma_i^+\sigma_j^- + \text{h.c})$ onto the two-band basis, we find that the interaction Hamiltonian reads

$$H^{\rm int} = \frac{J}{2}\sum_{i,j}\Big[V_a^{ij}\,\tilde\sigma_i^{aa}\tilde\sigma_j^{aa} + V_b^{ij}\,\tilde\sigma_i^{bb}\tilde\sigma_j^{bb} + V_{ab}^{ij}\big(\tilde\sigma_i^{aa}\tilde\sigma_j^{bb} + \tilde\sigma_i^{bb}\tilde\sigma_j^{aa}\big) + V_{ab}^{ij\,\prime}\,\tilde\sigma_i^{x}\tilde\sigma_j^{x} + \\ + V_{3ab}^{ij}\big(\tilde\sigma_i^{x}\tilde\sigma_j^{aa} + \tilde\sigma_i^{aa}\tilde\sigma_j^{x}\big) + V_{3ba}^{ij}\big(\tilde\sigma_i^{x}\tilde\sigma_j^{bb} + \tilde\sigma_i^{bb}\tilde\sigma_j^{x}\big)\Big](\sigma_i^+\sigma_j^- + \text{h.c}), \tag{35}$$

where we have introduced the operators $\tilde{\sigma}^{\alpha\alpha} = |\alpha\rangle\langle\alpha|$ and $\tilde{\sigma}_i^x = |a\rangle\langle b| + |b\rangle\langle a|$, acting on the motional basis. Because of the periodicity of the system, the Wannier wavefunction centered on site i, $w_{i,\alpha}(x)$ is equal to $w_{0,\alpha}(x - x_i)$. Thus the matrix elements appearing in Supplementary Equation (35) can be written as

$$V_\alpha^{ij} = \int dx dx' \, \sin kx \sin kx' \, e^{-|x_i - x_j - x + x'|/L} \, w_\alpha^2(x) w_\alpha^2(x'), \tag{36}$$

$$V_{ab}^{ij} = \int dx dx' \, \sin kx \sin kx' \, e^{-|x_i - x_j - x + x'|/L} \, w_a^2(x) w_b^2(x'), \tag{37}$$

$$V_{ab}^{ij\prime} = \int dx dx' \, \sin kx \sin kx' \, e^{-|x_i - x_j - x + x'|/L} \, w_a(x) w_b(x) w_a(x') w_b(x'), \tag{38}$$

$$V_{3\alpha\alpha'}^{ij} = \int dx dx' \, \sin kx \sin kx' \, e^{-|x_i - x_j - x + x'|/L} \, w_\alpha^2(x) w_\alpha(x') w_{\alpha'}(x'), \tag{39}$$

with the site dependence now only appearing in the exponential.

To simplify further the matrix elements we can use the initial assumption that the functions are tightly confined around the lattice sites and the the overlap between functions at different sites is negligible. Indeed, $|x - x'| < L$ in the region over which the wavefunctions will have appreciable weight, motivating an expansion of the coefficients in powers of $1/L$. Furthermore, we will assume that $L \sim a$, such that we can make the nearest-neighbor approximation for interactions. By exploiting the parity of the functions w_a and w_b and the sine function, one finally arrives at

$$H^{\text{int},(0)} = 2g \sum_i \tilde{\sigma}_i^x \tilde{\sigma}_{i+1}^x \left(\sigma_i^+ \sigma_{i+1}^- + \text{h.c.}\right), \tag{40}$$

$$H^{\text{int},(1)} = -2g(2a/L) \sum_i \left\{ \frac{\eta_a + \eta_b}{2\eta_0} \left(\tilde{\sigma}_i^x - \tilde{\sigma}_{i+1}^x\right) + \frac{\eta_b - \eta_a}{2\eta_0} \left(\tilde{\sigma}_i^x \tilde{\sigma}_{i+1}^z - \tilde{\sigma}_{i+1}^x \tilde{\sigma}_{i+1}^z\right) \right\} \left(\sigma_i^+ \sigma_{i+1}^- + \text{h.c.}\right), \tag{41}$$

where $H^{\text{int},(n)}$ denote the expansions of the Hamiltonian in powers of L^{-n}. Here we have defined $g = Je^{-2a/L}\,\eta_0^2/2$, $\eta_0 = \int dx \, \sin k_0 x \, w_a(x) w_b(x)$ and $\eta_{a,b} = (1/2a) \int dx \, x \sin k_0 x \, w_{a,b}^2(x)$. Hamiltonian (4) of the main text is given by the sum of Supplementary Equations (40), (41), $H^{\text{band}} = \Delta \sum_i \tilde{\sigma}_i^z$ and $H^{\text{magn}} = h \sum_i \sigma_i^z$.

In the case in which both the magnetic field h and the coupling constant g are much smaller than Δ, one can obtain an effective Hamiltonian for the low-energy physics by applying Schrieffer-Wolff (SW) transformations [9] on Hamiltonian (4) of the main text. Before applying the transformation it is convenient to write the spin operators as fermions via a Jordan-Wigner transformation, and the pseudo-spin as Holstein-Primakoff bosons:

$$\tilde{\sigma}_i^- = \sqrt{1 - b_i^\dagger b_i}\, b_i, \tag{42}$$

$$\tilde{\sigma}_i^+ = b_i^\dagger \sqrt{1 - b_i^\dagger b_i}, \tag{43}$$

$$\tilde{\sigma}_i^z = -1 + 2 b_i^\dagger b_i. \tag{44}$$

This transformation is particularly convenient when, as in our case, $\langle b_i^\dagger b_i \rangle \ll 1$, since the square root operators can be expanded. Retaining up to second order in the bosonic operators we have

$$H^{\text{wc}} = (-\Delta + h)N + \sum_i 2\Delta\, b_i^\dagger b_i - 2h\, c_i^\dagger c_i + \\ + 2g \left\{ (b_i^\dagger + b_i)(b_{i+1}^\dagger + b_{i+1}) - \chi(b_i^\dagger + b_i - b_{i+1}^\dagger - b_{i+1}) \right\} \left(c_i^\dagger c_{i+1} + \text{h.c.}\right), \tag{45}$$

where $\chi = \eta_a/(L\eta_0)$. The SW transformation is based on the division of the Hilbert space into a low-energy subspace, that one with zero bosonic excitations, whose projector is P_0, and a high-energy one containing excitations whose projector is Q_0. For Supplementary Equation (45) it is convenient to apply the transformation separately for the term linear in bosonic operators $V_\text{L} = -2g\chi(b_i^\dagger + b_i - b_{i+1}^\dagger - b_{i+1})$ and the term quadratic $V_\text{Q} = 2g(b_i^\dagger + b_i)(b_{i+1}^\dagger + b_{i+1})$. We will show explicitly how the transformation acts on the first one.

The effective Hamiltonian is given to the first order by

$$H_\text{L}^{\text{eff,wc}} = \mathcal{D}(V_\text{L}) + \frac{1}{2} P_0 [S, \mathcal{O}(V_\text{L})] P_0, \tag{46}$$

where the operators $\mathcal{D}(X) = P_0XP_0 + Q_0XQ_0$ and $\mathcal{O}(X) = P_0XQ_0 + Q_0XP_0$ take respectively the diagonal and the off-diagonal components of an operator (with respect to the low-energy and high-energy subspaces defined above), and

$$S = \sum_{p,q} \frac{\langle p|\,\mathcal{O}(V_{\rm L})\,|q\rangle}{E_p - E_q}\,|p\rangle\,\langle q|\,, \tag{47}$$

with $|p\rangle\,, |q\rangle$ belonging to different subspaces. Clearly $\mathcal{D}(V_{\rm L}) = 0$ and $\mathcal{O}(V_{\rm L}) = V_{\rm L}$ so that

$$\frac{1}{2}P_0[S, V_{\rm L}]P_0 = -\frac{(2g\chi)^2}{2\Delta}\sum_{q,q'}\langle q'|\sum_i\left(c_i^\dagger c_{i+1} - c_{i-1}^\dagger c_i + \text{h.c.}\right)^2|q\rangle\,|q'\rangle\,\langle q|\,. \tag{48}$$

Thus,

$$H_{\rm L}^{\rm eff,wc} = -\frac{(2g\chi)^2}{2\Delta}\sum_i\left(c_i^\dagger c_{i+1} - c_{i-1}^\dagger c_i + \text{h.c.}\right)^2 = \frac{(2g\chi)^2}{2\Delta}\sum_i 4c_i^\dagger c_i\big(c_{i+1}^\dagger c_{i+1} - 1\big) + \big(c_{i-1}^\dagger c_{i+1} + \text{h.c.}\big)\big(1 - 2c_i^\dagger c_i\big). \tag{49}$$

Performing a similar transformation on the quadratic Hamiltonian $V_{\rm Q}$ we obtain

$$H_{\rm Q}^{\rm eff,wc} = \frac{2g^2}{2\Delta}\sum_i c_i^\dagger c_i\big(c_{i+1}^\dagger c_{i+1} - 1\big). \tag{50}$$

Combining the two transformations, which commute, we obtain

$$H^{\rm eff,wc} = \sum_i h\left(1 - 2c_i^\dagger c_i\right) + \frac{2g^2(1+4\chi^2)}{\Delta}c_i^\dagger c_i\big(c_{i+1}^\dagger c_{i+1} - 1\big) + \frac{2g^2\chi^2}{\Delta}\big(c_{i-1}^\dagger c_{i+1} + \text{h.c.}\big)\big(1 - 2c_i^\dagger c_i\big), \tag{51}$$

which can be expressed by a JW transformation in terms of spin operators as Hamiltonian (5) of the main text.

We now discuss the limit of large magnetization. Hamiltonian (4) of the main text commutes with the total magnetization $M_z = (1/2)\sum_i \sigma_i^z$, but not with the operator $O = \sum_i \tilde{\sigma}_i^z\sigma_i^z$. The operator $O_i = \tilde{\sigma}_i^z\sigma_i^z$ has eigenvalues ± 1, each with two-fold degeneracy. It is useful to decompose the Hamiltonian into a part H' commuting with O and a part H'' that does not.

The part that commutes with O consists of the non interacting part of (4) as well as the $\tilde{\sigma}_i^x\tilde{\sigma}_{i+1}^x$ term in the interaction, and can be written as

$$\begin{aligned} H' = \sum_i 1/2\big(\Delta + h\big)\big(\tilde{\sigma}_i^z + \sigma_i^z\big) + 1/2\big(\Delta - h\big)\big(\tilde{\sigma}_i^z - \sigma_i^z\big) + \\ + 2g\big(\tilde{\sigma}_i^+\tilde{\sigma}_{i+1}^+ + \tilde{\sigma}_i^+\tilde{\sigma}_{i+1}^- + \tilde{\sigma}_i^-\tilde{\sigma}_{i+1}^+ + \tilde{\sigma}_i^-\tilde{\sigma}_{i+1}^-\big)\big(\sigma_i^+\sigma_{i+1}^- + \sigma_i^-\sigma_{i+1}^+\big). \end{aligned} \tag{52}$$

It is convenient to introduce the following two sets of operators

$$\tau_i^z = (\tilde{\sigma}_i^z + \sigma_i^z)/2 \tag{53}$$
$$\tau_i^+ = \tilde{\sigma}_i^+\sigma_i^+ \tag{54}$$
$$\tau_i^- = \tilde{\sigma}_i^-\sigma_i^- \tag{55}$$

and

$$\gamma_i^z = (-\tilde{\sigma}_i^z + \sigma_i^z)/2 \tag{56}$$
$$\gamma_i^+ = \tilde{\sigma}_i^-\sigma_i^+ \tag{57}$$
$$\gamma_i^- = \tilde{\sigma}_i^+\sigma_i^- \tag{58}$$

These two sets of operators obey the usual spin algebra, with operators from different sets commuting. The two sets of operators act on different subspaces of the local Hilbert space, corresponding to the two subspaces with eigenvalues ± 1 of the operators O_i, i.e. $\mathcal{H}_i = \mathcal{H}_i^+ \oplus \mathcal{H}_i^-$. We can thus write H' as

$$H' = H^+ + H^- + H^{+-}, \tag{59}$$

where

$$H^+ = \sum_i (\Delta + h)\tau_i^z + 2g(\tau_i^+\tau_{i+1}^- + \text{h.c.}), \tag{60}$$

$$H^- = \sum_i (-\Delta + h)\gamma_i^z + 2g(\gamma_i^+\gamma_{i+1}^- + \text{h.c.}) \tag{61}$$

and

$$H^{+-} = 2g\sum_i (\tau_i^+\gamma_{i+1}^- + \gamma_i^-\tau_{i+1}^+ + \gamma_i^+\tau_{i+1}^- + \tau_i^-\gamma_{i+1}^+). \tag{62}$$

H'' has a lengthy expression and will not be reproduced here.

As explained in the main text, when the system is nearly fully polarized (paramagnetic phase), with few spins flipped we have that H'' is much less relevant than H'. Note that the spins are prevalently in state $|\Downarrow\rangle = |a, \downarrow\rangle$, which belongs to the subspace described by H^+. One expects then that H^+ forms a good description of the system, with other terms in the Hamiltonian giving perturbative corrections. Note that H^+ connects the state $|\Downarrow\rangle = |a, \downarrow\rangle$ to $|\Uparrow\rangle = |b, \uparrow\rangle$, i.e. a simultaneous flip of the spin and band should occur.

Finally, we describe the criteria by which we numerically identify the phases shown in Fig. 3b of the main text. The border of the paramagnetic phase is immediately identified, since the phase consists of the points of the $g - h$ plane with magnetization per spin $M_z = -1/2$. Note that, given that M_z commutes with H and that the system is finite, the values of the total magnetization obtained numerically are integers, so that it is not necessary to define any tolerance for this order parameter.

As explained in the main text, to identify the Néel ordered phase "N" and the dimerized phase "D" we examine the values of their characteristic order parameters Φ and D_T. In particular, we attribute a point to "N" if $\Phi > 0.025$ and to "D" if $M_z = 0$ and $D_\text{T} > 0.01$. To avoid contributions from the edges of the chain we evaluate the order parameters only in the 20 central atoms of the chain, even when the "bulk" of the chain, i.e. the region where the expectation values of the observables are uniform with a certain period (2 for these two phases), is substantially more extended.

The lower border of the "SMF" phase can be identified by comparing the magnetization profile obtained with the DMRG with that one predicted by H^+. One can indeed identify a value of the magnetic field $\bar{h}(g)$ for which $\partial M_z/\partial h$ increases for decreasing h, in qualitative disagreement with the behaviour of M_z predicted by H^+. This happens at $M_z \approx -1/4$ and for $g \gtrsim 1.3\Delta$ the change in the magnetization curve is particularly sharp.

The borders of the trimerized phase "T" are determined by taking those of constant magnetization $M_z = -20/62 \sim -1/3$, and the condition that the expectation values of the observable are uniform with period 3 in the central region (20 atoms) of the chain, as in Fig. 5d of the main text. This happens for values of $g \gtrsim 1.3\Delta$, comparable with the coupling strength required for having dimerization.

Supplementary note 4. Three-band model of quantum motion

Here we present the results of numerical simulations where a third motional band is added. Specific values of observables and the phase boundaries quantitatively change. However, we find that the major conclusions presented before remain intact, and justify the two-band limit as a simple model that captures the important physics.

Under the same assumption of tight confinement of the atoms in the local traps, one can show that, up to first order in $1/L$ in the expansion of the exponential, the interaction Hamiltonian generalizes to

$$H^{\text{int},(0)} + H^{\text{int},(1)} = Je^{-2a/L}\sum_i \left[\tilde{\Theta}_i\tilde{\Theta}_{i+1} - (\tilde{\Theta}_i\tilde{\Phi}_{i+1} - \tilde{\Phi}_i\tilde{\Theta}_{i+1})\right](\sigma_i^+\sigma_{i+1}^- + \text{h.c}), \tag{63}$$

where

$$\tilde{\Theta}_i = \sum_{\alpha,\beta|p(\alpha)p(\beta)=-1} \eta_{\alpha\beta}\, S_i^{\alpha\beta}, \tag{64}$$

$$\tilde{\Phi}_i = \sum_{\alpha,\beta|p(\alpha)p(\beta)=1} (2a/L)\,\eta'_{\alpha\beta}\, S_i^{\alpha\beta}. \tag{65}$$

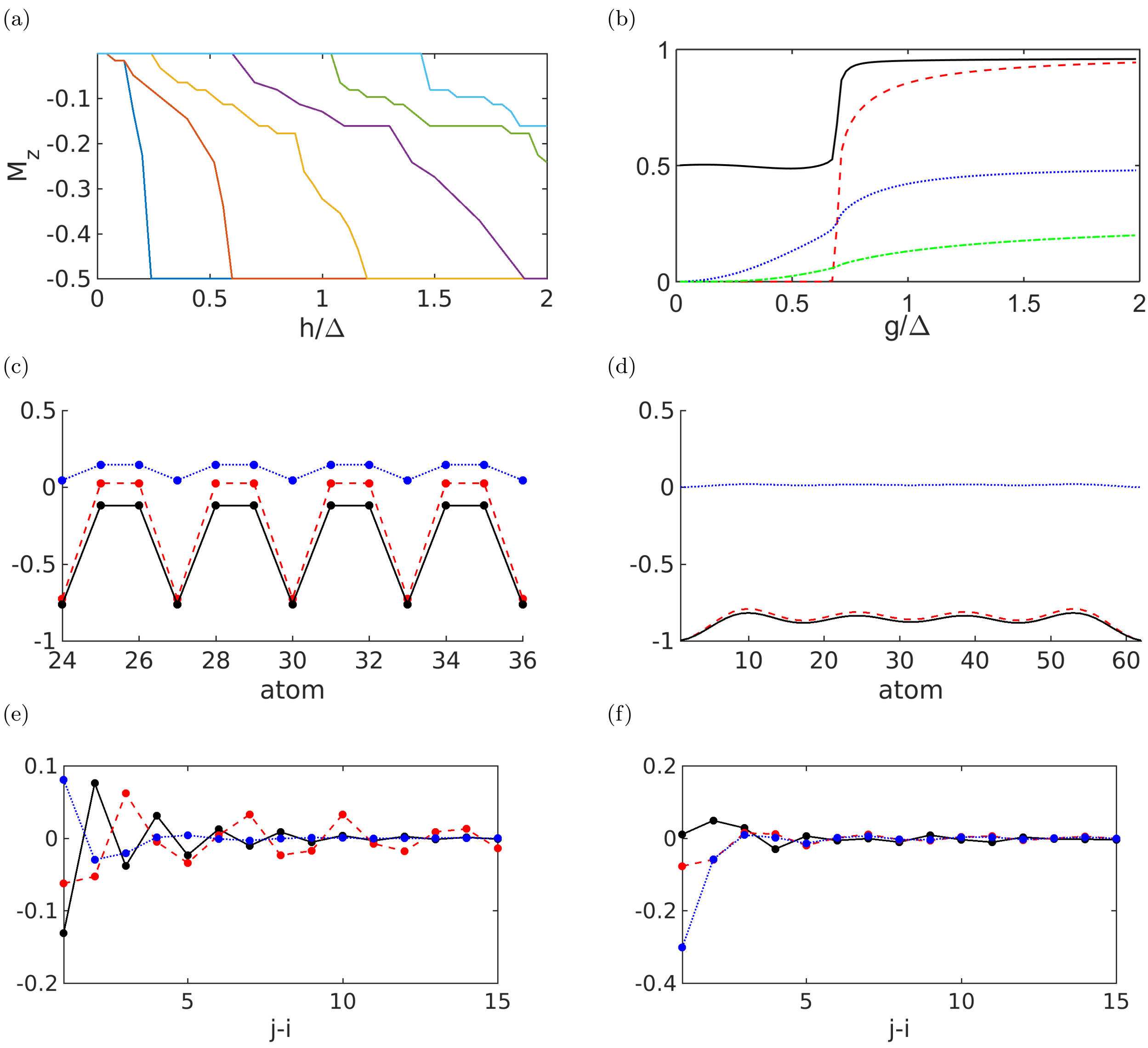

Supplementary Figure 2: **Correlation functions of the three-band model.** (a) Magnetization curve for different values of g ranging from 0.4Δ (dark blue) to 1.4Δ (light blue) as a function of h. (b) Triplet fraction (black solid), displacement (red dashed), second (blue dotted) and third band (green dot-dashed) population as function of g for $h = 0$ for the two atoms at the center of the chain. The displacement expectation value is normalized by the maximum eigenvalue of the position operator. (c) Three-band model: $\langle \sigma_i^z \rangle$ (black solid line), $\langle \tilde{\sigma}_i^z \rangle$, with $\tilde{\sigma}^z = \langle |b\rangle \langle b| - |a\rangle \langle a| \rangle$ (red dashed line), third band occupation $\langle |c\rangle \langle c| \rangle$ (blue dotted line) along the chain for the ground state at $(g, h) = (1.4, 1.6)\Delta$ belonging to the T phase. (d) As in (c) for the ground state at $(g, h) = (0.88, 1.42)\Delta$, in the SMF phase. (e)-(f) Same correlation functions of Figs. 5a-b of the main text but for the three band model at $(g, h) = (1.3, 2.2)\Delta$.

with $S^{\alpha\beta} = |\alpha\rangle \langle \beta|$, α, β being motional states and $p(\alpha), p(\beta)$ their parity, $\eta_{\alpha\beta} = \int dx \sin kx\, w_\alpha(x) w_\beta(x)$ and $\eta'_{\alpha\beta} = (1/a) \int dx\, x \sin kx\, w_\alpha(x) w_\beta(x)$. One can readily check that if we allow the sum on α, β to run over the first two motional states $|a\rangle$ and $|b\rangle$ we recover Supplementary Equations (40) and (41).

In the following we present some results obtained by studying numerically the ground state of the three-band Hamiltonian. The Wannier function $w_c(x)$ corresponding to the third band is numerically calculated for the same ratio of trap depth to recoil energy ($V_{\mathrm{L}}/E_{\mathrm{R}} = 20$) as in the two-band case. In Supplementary Figure 2a we plot the magnetization curves for different values of g ranging from 0.4 to 1.4 Δ. g is defined as before (i.e. involving only the Wannier functions of the first two bands). With respect to the two-band model we notice that there is a compression along the direction of changing g. We can explain this change by the fact that the atoms can displace more in the three-band model with the consequence of increasing the interaction energy with respect to the magnetic one. Nevertheless, we can observe in the magnetization curves the appearance of a plateau at $M_z = -1/6$ and the characteristic magnetization profile of the SMF phase.

In Supplementary Figure 2b, we consider the two atoms at the center of the chain, for zero magnetic field ($h = 0$)

and varying the interaction strength g. In particular, we plot the triplet fraction associated with the two-atom reduced density matrix, the (identical) displacements $\langle |x| \rangle$ from the trap center, and the (identical) populations of each atom in the second and third bands. Here, the displacement is normalized by the maximum value possible, within the set of wave functions composed of a superposition of Wannier functions of the three bands. We can see that after the transition to the dimerized phase, indicated by non-zero displacement, the displacement steadily increases and eventually saturates with increasing g. The same effect is observed for two bands (with a smaller allowed maximum value), and is clearly a result of the finite number of Wannier functions used. As no new phases appear when a third band is added, one can reasonably infer that adding progressively more bands simply would result in a greater displacement for very large values of g. The motion in the dimerized phase thus would appear "classical" (*e.g.* one could make an ansatz that the motional state is a coherent state), and this result would be consistent with the results presented in Sec. III of main text, where an increasing dimerization is seen when the atoms are treated as classical point particles.

In Supplementary Figures 2c-d we plot the expectation values of $\tilde{\sigma}^z$ (with definition unchanged from the two-band model) and σ^z for two points belonging to the trimerized and SMF phases, respectively. It can be see that the features that characterize these phases are not changed. In the same figures we also plot the population of the third band, which we find to be small in the first case and negligible in the second one. For the SMF phase this result was expected from the fact that already in the two-band model the population of the excited motional state was small. For the trimerized phase it was instead not obvious *a priori*, since in the two-band model the excited motional state was significantly populated.

Finally, in Supplementary Figures 2e-f we reproduce the two-band model correlation functions of Figs. 5a-b of the main text for the ground state at $(g, h) = (1.3, 2.2)\Delta$, this time including three bands. The values of (g, h) are different than those used in the main text, but are chosen such that the ground states have identical magnetization. We can see that the correlation function $\langle \tau_i^+ \tau_j^- \rangle - \langle \tau_i^+ \rangle \langle \tau_j^- \rangle$ remains regular (suggesting a Luttinger liquid description for these composite particles), while correlations involving the spin or motion alone display more erratic behavior, like before.

Supplementary References

[1] Buhmann, S. Y. & Welsch, D.-G., Dispersion forces in macroscopic quantum electrodynamics, Prog. Quantum Electron. **31**, 51 (2007).

[2] Dung, H. T., Knöll, L. & Welsch, D.-G., Resonant dipole-dipole interaction in the presence of dispersing and absorbing surroundings, Phys. Rev. A **66**, 063810 (2002).

[3] Goban, A. *et al.*, Atom–light interactions in photonic crystals, Nature Commun. **5**, 3808 (2014).

[4] Hood, J. D. *et al.*, Atom-atom interactions around the band edge of a photonic crystal waveguide, Proc. Natl. Acad. Sci. USA **113**, 10507 (2016).

[5] Douglas, J. S. *et al.*, Quantum many-body models with cold atoms coupled to photonic crystals, Nature Photon. **9**, 326 (2015).

[6] Goldstein, E. V. & Meystre, P., Dipole-dipole interaction in optical cavities, Phys. Rev. A **56**, 5135 (1997).

[7] Asenjo-Garcia, A., Hood, J. D., Chang, D. E. & Kimble, H. J., Atom-light interactions in quasi-1D nanostructures: a Green's function perspective, preprint at https://arxiv.org/abs/1606.04977 (2016).

[8] Jordan, P. & Wigner, E., Über das Paulische Äquivalenzverbot, Z. Physik **47**, 631 (1928).

[9] Bravyi, S., DiVincenzo, D. P. & Loss, D., Schrieffer–Wolff transformation for quantum many-body systems, Ann. Phys. **326**, 2793 (2011).
